# Y705 and S727 are required for the mitochondrial import and transcriptional activities of STAT3, and for regulation of stem cell proliferation

**DOI:** 10.1242/dev.199477

**Published:** 2021-09-06

**Authors:** Margherita Peron, Alberto Dinarello, Giacomo Meneghetti, Laura Martorano, Riccardo M. Betto, Nicola Facchinello, Annachiara Tesoriere, Natascia Tiso, Graziano Martello, Francesco Argenton

**Affiliations:** 1 Department of Biology, University of Padova, 35121, Padova, Italy; 2 Department of Molecular Medicine, University of Padova, 35121, Padova, Italy

**Keywords:** Embryonic stem cells, STAT3, Mitochondria, Transcription, Zebrafish

## Abstract

The STAT3 transcription factor, acting both in the nucleus and mitochondria, maintains embryonic stem cell pluripotency and promotes their proliferation. In this work, using zebrafish, we determined *in vivo* that mitochondrial STAT3 regulates mtDNA transcription in embryonic and larval stem cell niches and that this activity affects their proliferation rates. As a result, we demonstrated that import of STAT3 inside mitochondria requires Y705 phosphorylation by Jak, whereas its mitochondrial transcriptional activity, as well as its effect on proliferation, depends on the MAPK target S727. These data were confirmed using mouse embryonic stem cells: although the Y705-mutated STAT3 cannot enter mitochondria, the S727 mutation does not affect import into the organelle and is responsible for STAT3-dependent mitochondrial transcription. Surprisingly, STAT3-dependent increase of mitochondrial transcription appears to be independent from STAT3 binding to STAT3-responsive elements. Finally, loss-of-function experiments, with chemical inhibition of the JAK/STAT3 pathway or genetic ablation of *stat3* gene, demonstrated that STAT3 is also required for cell proliferation in the intestine of zebrafish.

## Abstract (precis)

**Summary:** Mitochondrial import of STAT3 requires Y705 phosphorylation by Jak, whereas STAT3 mitochondrial transcriptional activity and its effect on proliferation depend on the MAPK target S727.

## INTRODUCTION

Many of the major human malignancies display elevated levels of constitutively activated STAT3 (Johnston and Grandis, 2011; O'Shea et al., 2015). Most interestingly, recent data report that STAT3 target genes are overexpressed in tumour-initiating cancer stem cells (Fouse and Costello, 2013; Wei et al., 2014; Ghoshal et al., 2016). Stat3 is also the key mediator of leukemia inhibitory factor (LIF) in mouse embryonic stem cells (ESCs), in which the LIF/STAT3 axis promotes the maintenance and induction of naïve pluripotency (Burdon et al., 1999; Matsuda et al., 1999; Smith et al., 1988; Martello et al., 2013).

STAT3 transcriptional activity is regulated by phosphorylation of two separated residues. When Janus kinases 1/2/3 (JAK1/2/3) phosphorylate its tyrosine 705 (Y705), STAT3 homodimerises, enters the nucleus, binds to STAT3 response elements and triggers transcription of its target genes (Ni et al., 2004). On the other hand, the function of serine 727 (S727) phosphorylation remains controversial; pS727 has been reported to have both activating and inhibitory effects on STAT3 transcriptional activity (Qin et al., 2008; Shi et al., 2006). More recently, it has been demonstrated that pY705 is absolutely required for STAT3-mediated ESCs self-renewal, whereas pS727 is dispensable, serving only to promote proliferation and optimal pluripotency (Huang et al., 2014). Notably, zebrafish mutants lacking maternal and zygotic Stat3 expression display transient axis elongation defects due to reduced cell proliferation during embryogenesis (Liu et al., 2017). In addition, it has been recently demonstrated in zebrafish that Stat3 is transcriptionally active in the stem cells of highly proliferative tissues like tectum opticum (TeO), hematopoietic tissue and intestine (Peron et al., 2020).

Notably, a pool of STAT3 has been revealed inside the mitochondria of different cell types, placing this transcription factor in the large family of dual-targeted proteins with both nuclear and mitochondrial functions (Wegrzyn et al., 2009; Mantel et al., 2012). Another recently discovered subcellular localisation of STAT3 is the endoplasmic reticulum (ER), in which STAT3 controls the release of Ca^2+^, with consequences on the mitochondrial Ca^2+^ levels and on life-death cell-fate decisions. This function is crucial for the maintenance of apoptosis-resistant cells in the tumour niche (Avalle et al., 2019). Although the mechanisms of action of mitochondrial STAT3 (mitoSTAT3) are still debated, it is calculated that 10-25% of total cellular STAT3 is in the mitochondria (Szczepanek et al., 2011; Carbognin et al., 2016). By using different *in vitro* models to infer its function, several roles have been proposed for mitoSTAT3: interaction with mitochondrial respiratory chain complexes I and II; binding to the D-Loop regulatory region of mitochondrial DNA (mtDNA); regulation of mitochondrial gene expression; and regulation of the mitochondrial permeability transition pore (Wegrzyn et al., 2009; Macias et al., 2014; Meier and Larner, 2014; Carbognin et al., 2016). However, the molecular mechanisms leading to mitoSTAT3 activation and translocation are only partially understood. Previous *in vitro* studies have suggested that the S727 phosphorylation by MAPK kinases may be required for STAT3 mitochondrial activity (Gough et al., 2013) and is needed to restore the activity of complexes I and II in *Stat3*^−/−^ cells (Wegrzyn et al., 2009). Moreover, pS727 STAT3, when targeted to the mitochondria, is able to promote Ras-dependent transformation of human bladder carcinoma cells (Gough et al., 2009). Notably, the role of other post-translational modifications on mitoSTAT3 activities has not been investigated yet.

Zebrafish is an organism widely used for analysis of gene expression and protein-gene functions during development, mostly because of its transparency and external fertilisation. Starting from the observation that STAT3 and its functional domains are highly conserved in zebrafish (Liang et al., 2012; Oates et al., 1999) we used this animal model to study the mitoSTAT3 pathway *in vivo*. In this paper we demonstrate that mitoSTAT3 mitochondrial function relies on phosphorylation of both Y705 and S727 by JAK1/2/3 and ERK kinases, respectively. Data also show that mitoSTAT3 modulation of mitochondrial transcription does not require a canonical STAT3 DNA binding domain, with the differences between the eukaryotic and the prokaryotic transcriptional machineries operating in the nucleus and mitochondria, respectively. Finally, using zebrafish larvae, we directly linked the STAT3-dependent regulation of tissue stem cells proliferation to mitochondrial transcriptional activity.

## RESULTS

### mitoSTAT3 regulates cell proliferation in the PML of the TeO through mtDNA transcription

**Fig. 1.**
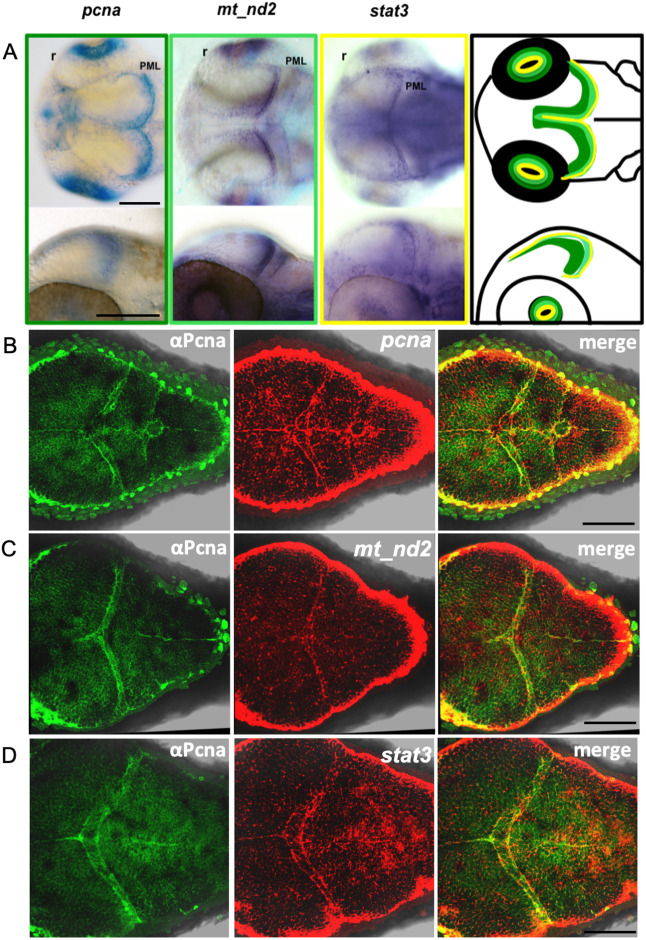
***Stat3* mRNA is co-expressed with proliferation and mtDNA transcription markers in the TeO of zebrafish embryos.** (A) WISH on 48 hpf zebrafish WT embryos using *pcna* (dark green frame and outline), *mt_nd2* (light green frame and outline) and *stat3* (yellow frame and outline) antisense mRNA probes shows co-expression of the three transcripts in the PML region of the TeO. PML, peripheral midbrain layer; r, retina. (B) IF against Pcna (green) and FISH with *pcna* antisense mRNA probe (red). (C) IF against Pcna (green) and FISH with *mt_nd2* antisense mRNA probe (red). (D) IF against Pcna (green) and FISH with *stat3* antisense mRNA probe (red). Images are representative of the 90% of processed samples. Scale bars: 100 μm.

**Fig. 2.**
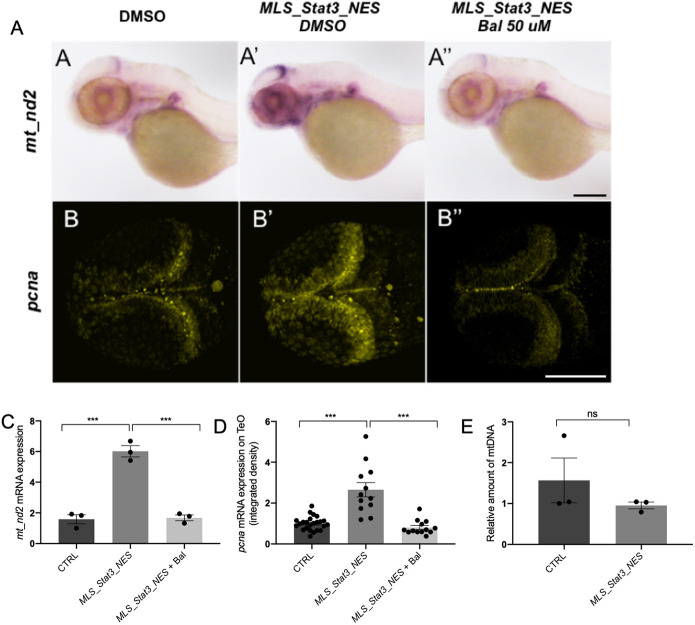
**mitoSTAT3 regulates proliferation through mitochondrial DNA transcription.** (A-A″) WISH with anti-*mt_nd2* mRNA probe representing mitochondrial gene transcription in uninjected embryos (A), embryos injected with *MLS_mStat3_NES* mRNA (A′) and injected embryos treated with 50 µM Balapiravir (A″). (B-B″) FISH with *pcna* probe in the TeO of uninjected embryos (B), embryos injected with *MLS_mStat3_NES* mRNA (B′) and injected embryos treated with 50 µM Balapiravir (B″). (C) qRT-PCR showing *mt_nd2* gene expression after injection of *MLS_mStat3_NES* mRNA and treatment with Balapiravir at 48 h post injection; *zgapdh* was used as internal control. (D) Fluorescence quantification of *pcna* mRNA expression in the TeO (*n*=12). (E) Relative amount of mtDNA in embryos injected with *MLS_mStat3_NES* mRNA and uninjected controls at 48 hpf. Mean dCt values were calculated as Ct of *mt_nd1* (mitochondrial-encoded gene) minus Ct of *polg1* (nuclear-encoded gene). Data are mean±s.e.m. *P<0.05; **P<0.01; ***P<0.001 (unpaired two-tailed *t*-test on three independent biological samples, where *n* not specified)*.* ns, not significant. Scale bars: 200 μm in A; 100 μm in B.

It is known that in keratinocytes and mouse ESCs a portion of STAT3 localises to mitochondria, in which it induces mitochondrial transcription and cell proliferation (Macias et al., 2014; Carbognin et al., 2016). Therefore, we tested whether STAT3 and mitochondrial transcribed genes colocalise in proliferating regions of the zebrafish embryo, considering this colocalisation essential for STAT3-mitochondrial-proliferation liaison. Facilitated by the fact that *mt_nd2* expression profile has already been described in zebrafish (https://zfin.org/ZDB-PUB-010810-1) and considering the polycistronic nature of mtDNA-transcribed genes that results in stoichiometric mtDNA transcription (Taanman, 1999), we decided to use this mitochondrial gene as a hallmark of global mitochondrial gene expression. As described in Fig. 1, *mt_nd2* and the cellular proliferation-associated marker *pcna* are particularly expressed in regions also labelled by *stat3* transcripts, such as the inner retina and the peripheral midbrain layer (PML) of the TeO (Fig. 1A; Fig. 2A), the progenitor source for tectal and torus neurons in the embryo (Galant et al., 2016). To further visualise the link between *stat3* expression, mitochondrial transcription and cell proliferation, we performed immunofluorescence (IF) analysis of 48 h post fertilisation (hpf) larvae against Pcna protein and, subsequently, we performed *in situ* hybridisation (ISH) using RNA probes to detect *pcna*, *stat3* and *mt_nd2* mRNAs. In Fig. 1B we show that Pcna protein and *pcna* mRNA perfectly colocalise, demonstrating that both of these stainings can be used as reliable proliferation markers. Moreover, we demonstrate that Pcna protein expression and *mt_nd2* completely colocalise in the TeO at this developmental stage (Fig. 1C) and that *stat3* mRNA partially overlaps with Pcna (Fig. 1D).

In order to understand whether STAT3 mitochondrial activity regulates mtDNA transcription and promotes proliferation also *in vivo*, we injected zebrafish fertilised eggs with mRNA of a mitochondria-targeted murine form of *Stat3* (*mStat3*), provided with a nuclear export domain that makes it unable to localise to the nucleus (*MLS_mStat3_NES*) (Fig. S1A). This chimeric protein is completely devoid of nuclear functions as assessed by quantitative real time reverse transcription PCR (qRT-PCR) analysis of *Socs3*, the most direct *Stat3* target gene (Fig. S1B), and efficiently localises only inside the mitochondrion, as revealed by its colocalisation with the mitochondrial marker ATAD3, both in transfected mouse ESCs, and in zebrafish cells (Fig. S1C; Fig. S2A,B).

When *MLS_mStat3_NES* mRNA was injected into zebrafish embryos we could observe that this modified form of Stat3 was unable to induce the expression of its canonical target gene *socs3a* (Fig. S3A); however, we detected, both by ISH and qRT-PCR, a significant increase of mitochondrial transcription at 24 and 48 hpf (Fig. 2A,A′,C; Fig. S3B,D,E). Moreover, mitoSTAT3 does not affect the expression of *p53* (also known as *tp53*) mRNA in zebrafish larvae, demonstrating that *MLS_Stat3_NES* injection does not induce harmful effects in cellular homeostasis (Fig. S3C). It is worth noting that, as assayed by *pcna* analysis, overexpression of *MLS_mStat3_NES* mRNA also induced a proportional increase of this proliferation marker in the same tissues in which mitochondrial transcription was stimulated, i.e. the PML (Fig. 2B,D). On the other hand, we did not find any difference in mtDNA content when comparing injected and control embryos, suggesting that the effect of mitoSTAT3 on mitochondrial transcription is not due to increased mtDNA replication or mitochondrial biogenesis (Fig. 2E).

Importantly, chemical inhibition of mitochondrial transcription by Balapiravir (Feng et al., 2016) was able to abolish the effects of *MLS_Stat3_NES* on proliferation (Fig. 2A,B,C,D), thus providing evidence, *in vivo,* that the expression of the proliferation marker Pcna and of mitochondrial transcripts in the developing TeO of zebrafish embryos depends on mitoSTAT3-driven expression of mitochondrial genes.

### Mitochondrial STAT3 transcriptional activity relies on phosphorylation of both S727 and Y705

**Fig. 3.**
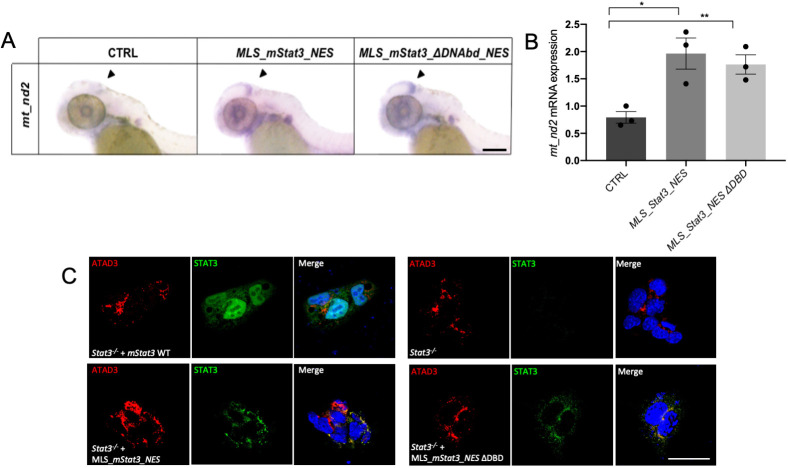
**Mutation of Stat3 DBD (DNA binding domain) does not affect its mitochondrial activities.** (A) WISH with anti-*mt_nd2* antisense probe in uninjected 48 hpf larvae and in 48 hpf larvae injected with *MLS_mStat3_NES* or *MLS_mStat3_ΔDNAbd_NES* mRNA. (B) qRT-PCR showing *mt_nd2* gene expression after injection of *MLS_mStat3_NES* or *MLS_mStat3_ΔDNAbd_NES* mRNA in 48 hpf embryos; *zgapdh* was used as internal control. (C) IF on ESCs transiently transfected with *MLS_mStat3_NES* or *MLS_mStat3_ΔDNAbd_NES* and stained with anti-STAT3 (green), anti-ATAD3 (red) antibodies and DAPI (blue). Data are mean±s.e.m. *P<0.05; **P<0.01 (unpaired two-tailed *t*-test on three independent biological samples, where *n* not specified). Scale bars: 200 μm.

To dissect the protein domains of STAT3 needed for the activation of mtDNA transcription, we focussed our experiments on the DNA binding and the transactivation domains. Putative STAT3 binding elements (SBE) have been identified in the mitochondrial D-Loop (the mitochondrial transcriptional initiation site) (Macias et al., 2014) and STAT3 was found to immunoprecipitate together with the mitochondrial D-Loop in mouse ESCs (Carbognin et al., 2016). Nonetheless, when a mitochondria-targeted form of STAT3 mutated in the DNA binding domain (STAT3 458-466 VVV-AAA; MLS_mStat3_ΔDNAbd_NES), and so unable to bind SBE (Horvath et al., 1995), was injected into zebrafish embryos, it retained its ability to activate *mt_nd2* transcription at levels comparable with those of wild-type (WT) mitoSTAT3 (Fig. 3A,B). It is worth noting that when transfected in mouse ESCs, this DNA binding mutant is still able to colocalise with ATAD3, a mitochondrial nucleoid marker (Fig. 3C). This result suggests that binding of STAT3 to SBE is dispensable for mtDNA transcription.

**Fig. 4.**
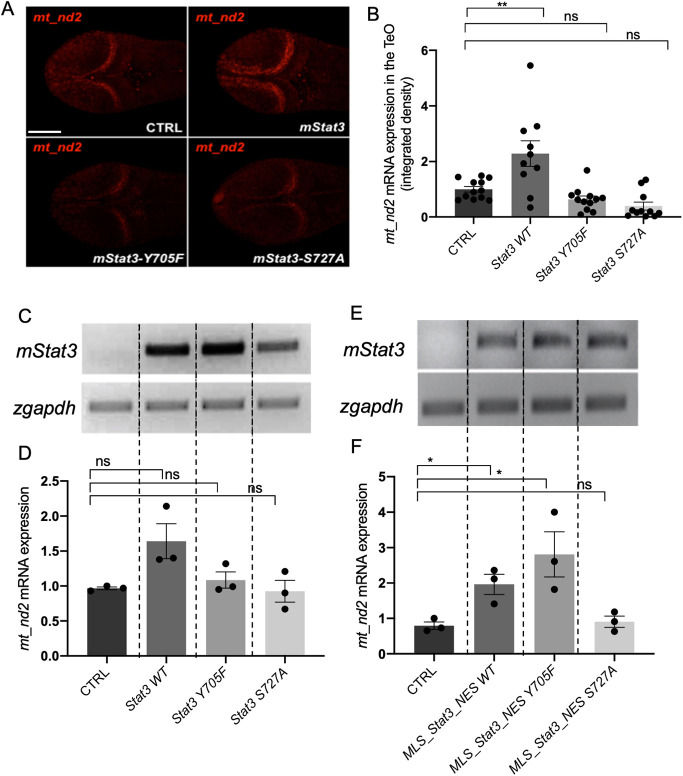
**mitoSTAT3 transcriptional activity relies on both S727 and Y705 phosphorylation.** (A) FISH with *mt_nd2* probe in the TeO of 48 hpf embryos injected with mRNA encoding the indicated mutated forms of *mStat3*. (B) Fluorescence quantification of *mt_nd2* mRNA expression in the TeO (*n*=10). (C) RT-PCR analysis of *mStat3* transcripts detected at 48 hpf in embryos injected with the indicated form of *mStat3* mRNA; *zgapdh* was used as internal control. (D) qRT-PCR analysis of *mt_nd2* transcript levels at 48 hpf normalised to *zgapdh*. (E) RT-PCR analysis of *MLS_mStat3_NES* transcripts detected at 48 hpf in embryos injected with indicated form of mitochondria-targeted *mStat3* mRNA; *zgapdh* was used as internal control. (F) qRT-PCR analysis of *mt_nd2* transcript levels at 48 hpf normalised to *zgapdh*. Data are mean±s.e.m. *P<0.05; **P<0.01 (unpaired two-tailed *t*-test on three independent biological samples, where *n* not specified)*.* ns, not significant. Scale bar: 100 μm.

It is known that JAK1/2/3-mediated phosphorylation on Y705 controls the nuclear activity of STAT3 and also ensures the stability of STAT3 monomers in the cytoplasm (Becker et al., 1998). On the other hand, phosphorylation on STAT3 S727 by the MAPK pathway (Ras-Raf-MEK-ERK pathway) is known, from *in vitro* studies, to enhance the electron transport chain (ETC) (Wegrzyn et al., 2009) as well as to promote cell proliferation and optimal pluripotency (Huang et al., 2014). To verify *in vivo* the post-translational requirements, and to test whether mitoSTAT3 activity requires Y705 phosphorylation, we decided to inject one-cell stage zebrafish embryos with mRNAs encoding mutant forms of murine *Stat3*. Specifically, we compared the activity of WT STAT3 [without the mitochondrial localisation sequence (MLS)] with two mutated forms, Y705F and S727A, able to prevent phosphorylation of residues 705 and 727, respectively (Minami et al., 1996; Mohr et al., 2013; Huang et al., 2014). Interestingly, when embryos were injected with the WT isoform, quantitative analysis of fluorescent *in situ* hybridisation (FISH) revealed a significant increase of mitochondrial transcription in the PML of the TeO (Fig. 4A,B). Increased expression was also noted in qRT-PCR experiments on whole embryos, but this did not reach statistical significance (Fig. 4C,D). Notably, when injecting either Y705F or S727A isoforms of STAT3, no stimulation of mitochondrial transcription in the PML population was detected, either using ISH or qRT-PCR (Fig. 4A-D; Fig. S4A). In conclusion, phosphorylation of both residues is needed for STAT3-mediated increase of mtDNA transcription in the PML. On the other hand, when the mutated isoforms are forcedly targeted to the mitochondrion [by using both the MLS and the nuclear export signal (NES)], the S727A mutation prevented mitoSTAT3-mediated activation of *mt_nd2* gene expression, whereas the STAT3-Y705F mutated isoform retained its mitochondrial transcriptional activity (Fig. 4E,F; Fig. S4B). This finding implies that Y705 is not directly involved in mitochondrial transcription.

**Fig. 5.**
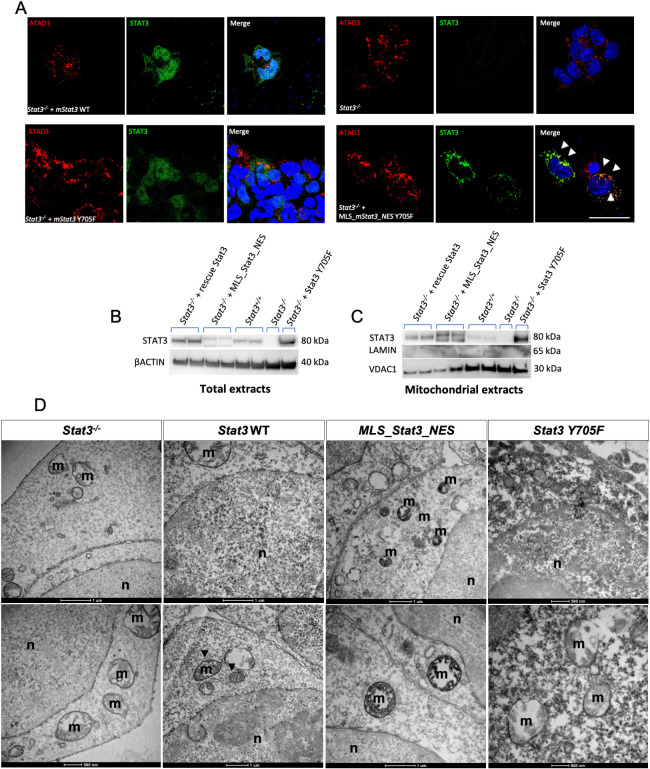
**Y705 phosphorylation is needed for the correct localisation of STAT3 in the mitochondrion.** (A) IF with anti-STAT3 and anti-ATAD3 antibodies on ESCs transiently transfected with either *mStat3*, *mStat3 Y705F* or *MLS_mStat3_NES Y705F*. Arrowheads indicate the colocalisation of ATAD and STAT3. (B) Western blot of total STAT3 in ESC extracts; β-actin was used as a loading control. (C) Western blot of mitochondrial STAT3 from ESC mitochondrial extracts; VDAC1 was used as a mitochondrial loading control, Lamin was used as a control for nuclear contamination. (D) Representative pictures of DAB IHC on ESCs acquired with TEM; positive signal is black and negative is white. Cristae are positive in *Stat3* WT and *MLS_Stat3_NES* transfected cells. m, mitochondria; n, nucleus. Scale bar: 200 μm (A).

Given that STAT3 Y705F had no direct effect on mitochondrial transcription (Fig. 4), we hypothesised that the tyrosine 705 could regulate STAT3 localisation. To further elucidate the localisation of mutated STAT3, we performed IF analysis on mouse *Stat3^−/−^* ESCs transiently transfected. Although transient transfection of STAT3-Y705F resulted in its nuclear and sporadic mitochondrial localisation, transfected mitoSTAT3-Y705F localised exclusively to mitochondria (Fig. 5A). Surprisingly, upon isolation of mitochondrial fractions followed by western blot analysis we could detect STAT3-Y705F, as well as WT STAT3, in mitochondria (Fig. 5B,C). Nonetheless, transmission electron microscopy (TEM) analysis after 3,3′-diaminobenzidine (DAB) immunohistochemistry (IHC) revealed that the accumulation of the staining was more localised in the nucleus in ESCs transfected with WT STAT3, whereas MLS_STAT3_NES localised inside mitochondria, staining cristae of the inner mitochondrial membrane (Fig. 5D). Of note, STAT3 Y705F forms clots along the edges of mitochondria, displays a diffuse cytoplasmic signal and fails to migrate to the intermembrane space, thus confirming that Y705 is essential for the correct localisation of STAT3 inside the mitochondrion.

### STAT3 S727 phosphorylation is needed for mitoSTAT3-driven promotion of cell proliferation in the PML

**Fig. 6.**
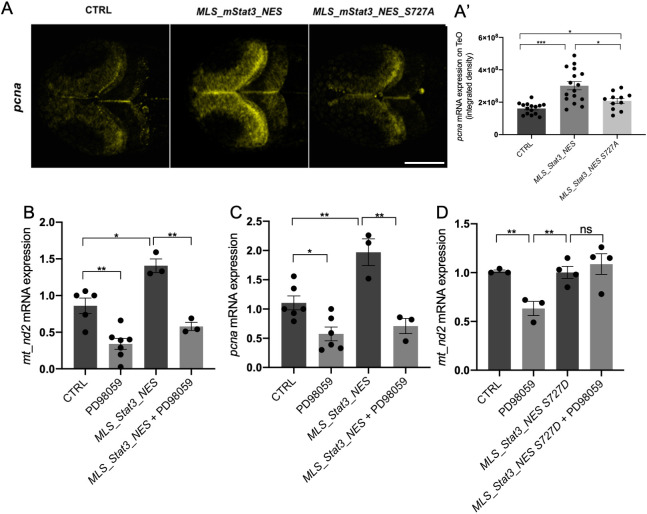
**mitoSTAT3-dependent activation of cell proliferation in the TeO depends on S727 phosphorylation.** (A,A′) Representative images of WISH performed with an anti-*pcna* probe on 48 hpf uninjected larvae, larvae injected with *MLS_mStat3_NES* or *MLS_mStat3_NES S727A* mRNAs (A). Fluorescence quantification of *pcna* mRNA expression in the TeO (*n*=12) (A′). (B) qRT-PCR analysis of *mt_nd2* in uninjected 48 hpf larvae and larvae injected with *MLS_mStat3_NES* mRNA treated or untreated with PD98059 for 24 h. (C) qRT-PCR analysis of *pcna* in uninjected 48 hpf larvae and larvae injected with *MLS_mStat3_NES* mRNA treated either with PD98059 12.5 µM or DMSO for 24 h. (D) qRT-PCR analysis of *mt_nd2* in uninjected 48-hpf larvae and larvae injected with *MLS_mStat3_NES S727D* mRNA treated or untreated with PD98059 for 24 h. actb1 was used as an internal control. Data are mean±s.e.m. *P<0.05; **P<0.01; ***P<0.001 (unpaired two-tailed *t*-test on three independent biological samples, where *n* not specified). ns, not significant. Scale bar: 100 μm.

As phosphorylation of S727 is needed for mitoSTAT3-driven mtDNA transcription, we tested whether this post-translational modification is also required for the increased expression of the proliferation marker Pcna in the PML. The expression of the proliferation marker Pcna in the PML of 48 hpf embryos injected with *MLS_mStat3_NES_S727A* mRNA was significantly lower than that of embryos injected with WT *MLS_mStat3_NES* mRNA (Fig. 6A), indicating that S727 phosphorylation is crucial for proliferation in the PML. Moreover, we performed IF and western blot analysis on *Stat3^−/−^* mouse ESCs transiently expressing either WT STAT3, STAT3 S727A or MLS_STAT3_NES, which showed that mitochondrial localisation of STAT3 is not affected by the S727A mutation (Fig. S5A,B).

On the other hand, it is known that in mouse 3T3 fibroblasts the MEK-ERK pathway is responsible for phosphorylation of STAT3 S727 (Gough et al., 2013). In order to evaluate *in vivo* the involvement of the MAPK pathway in phosphorylation of S727, and its downstream effects for mitoSTAT3-driven cell proliferation, we decided to use the MEK kinases inhibitor PD98059 (Alessi et al., 1995) commonly used *in vitro* to prevent S727 phosphorylation (Tian and An, 2004; Wang et al., 2020). First, we tested this compound in 3T3 mouse fibroblasts by checking the levels of STAT3 pS727 with western blot. Once we confirmed that PD98059 reduced the phosphorylation of S727 in 3T3 cells (Fig. S5C), we administered this compound to zebrafish embryos. Interestingly, WT larvae treated from 24 to 48 hpf with PD98059 displayed a significant reduction of *mt_nd2* and *pcna* transcript levels, suggesting that MEK inhibition reduced the expression of mitochondrial genes and the proliferation marker *pcna* (Fig. 6B,C). Of note, PD98059 is able to significantly block the positive effect of *MLS_mStat3_NES* injection: as shown in Fig. 6B and C, *MLS_mStat3_NES* injection results in an upregulation of *mt_nd2* and *pcna* expression. However, when *MLS_mStat3_NES*-injected larvae are treated with PD98059, there is a significant downregulation of *mt_nd2* and *pcna* mRNA expression compared with injected untreated siblings. These results demonstrate that PD98059 specifically affects mitochondrial activities of Stat3.

To further elucidate whether PD98059 was indeed targeting STAT3 S727, we decided to inject a mutated form of mitochondria-targeted *Stat3* (called *MLS_mStat3_NES S727D*) in which the S727 is substituted with aspartic acid. This modification has been already classified as phosphomimetic (Kim et al., 2009). As shown in Fig. 6D, PD98059 does not affect *mt_nd2* expression in larvae injected with *MLS_Stat3_NES S727D* mRNA.

In conclusion, S727 phosphorylation connects transcription of mitochondrial and proliferation genes; inhibitors of the MEK-ERK pathway affecting S727 phosphorylation, such as PD98059, abrogate both processes.

### Jak kinases maintain normal mtDNA transcription and proliferation in the PML and the intestine

**Fig. 7.**
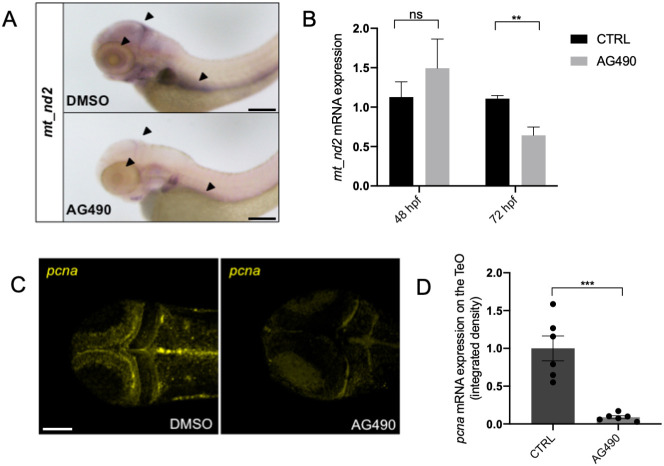
**JAK inhibition impairs normal mitochondrial transcription and cell proliferation in the TeO of 72** **hpf embryos.** (A) WISH with anti-*mt_nd2* mRNA probe on 72 hpf embryos treated with 100 µM AG490 from 24-72 hpf and DMSO-treated controls. (B) Relative *mt_nd2* transcript expression assayed by qRT-PCR in 48 and 72 hpf embryos treated with 100 µM AG490 and DMSO-treated controls starting from 24 hpf; *zgapdh* was used as internal control (*P*-values=0.6261; 0.0060). (C) FISH with anti-*pcna* probe in the TeO of 72 hpf embryos treated with 100 µM AG490 from 24 to 72 hpf and DMSO-treated controls. (D) Fluorescence quantification of *pcna* mRNA expression in the TeO (*n*=6) (*P*-value=0.0003). Data are mean±s.e.m. **P<0.01; ***P<0.001 (unpaired two-tailed *t*-test on three independent biological samples, where *n* not specified)*.* ns, not significant. Scale bars: 200 μm in A; 100 μm in C.

After demonstrating that STAT3-driven mitochondrial transcription relies on phosphorylation of both Y705 and S727, and that the consequential proliferation effect downstream of mitochondrial transcription requires functional MEK kinases, we decided to test the involvement of Jak Tyrosine-kinase activity, which promotes Y705 phosphorylation. WT embryos were treated from 24 to 72 hpf with AG490, a specific inhibitor of Jak2 widely used as a JAK/STAT3 inhibitor (Park et al., 2014; Gurbuz et al., 2014), and the expression of *mt_nd2* was assessed by qRT-PCR. When observed at 72 hpf, AG490-treated larvae displayed a significant reduction of *mt_nd2* expression in the PML, the inner retina and the primordium of the intestine (Fig. 7A, arrowheads), whereas no significant decrease was revealed at 48 hpf (Fig. 7B; Fig. S6A). In addition, proliferation-related activity was found to be significantly reduced in the PML of 72-hpf AG490-treated larvae, as assayed by ISH using anti-*pcna* probe (Fig. 7C,D).

**Fig. 8.**
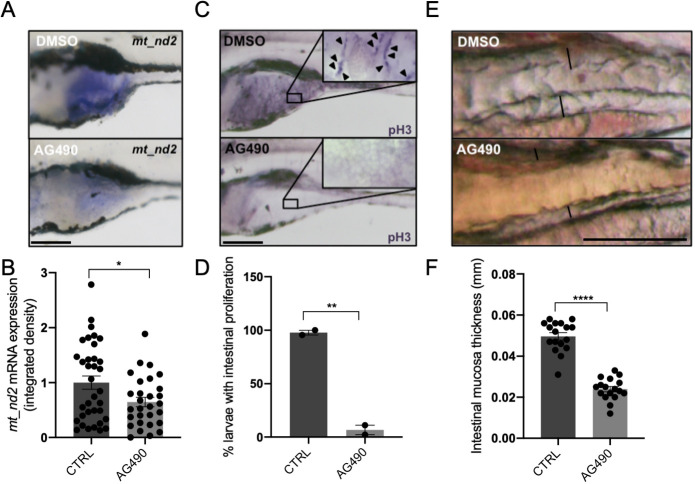
**JAK inhibition impairs normal mitochondrial transcription and cell proliferation in the intestine of 6** **dpf larvae.** (A) WISH with anti-*mt_nd2* mRNA probe on 6 dpf larvae treated with 60 µM AG490 from 24 to 72 hpf and DMSO-treated controls; zoom on the intestine. (B) Quantification of *mt_nd2* mRNA expression in the intestine (*n*=30). (C) Phospho-Histone-H3 (pH3) immunostaining of 6 dpf AG490-treated larvae and DMSO-treated controls; inset shows magnification of boxed area of intestine. Arrowheads indicate pH3-positive cells. (D) Quantification of the number of AG490- and DMSO-treated larvae displaying intestinal proliferation (*n*=15). (E) AG490-treated larvae showing loss of folding in intestinal mucosa. (F) Graph showing the dimension of mucosal thickness in both DMSO- and AG490-treated 6 dpf larvae (*n*=18). Data are mean±s.e.m. *P<0.05; **P<0.01; ***P<0.001 (unpaired two-tailed *t*-test on indicated number of samples). Scale bars: 200 μm in A,C; 100 μm in E.

We also investigated the effect of Jak inhibition in the intestine, a highly proliferating tissue of zebrafish larvae, in which the *mt_nd2* gene is strongly expressed between 3 and 6 days post fertilisation (dpf) (Fig. 8A). The activity of Stat3 in the intestine of zebrafish is consistent with the facts that: (1) the proliferative and survival effects of IL6 in murine intestinal epithelial cells (IECs) is largely mediated by STAT3 (Grivennikov et al., 2009); (2) STAT3 is needed for small-intestine crypt stem cell survival, as revealed by conditional mutant mice (Matthews et al., 2011); (3) Stat3-positive cells in zebrafish intestine represent a population of intestinal Wnt-responsive stem cells (Peron et al., 2020). The administration of 60 µM AG490 between 3 and 6 dpf was able to significantly reduce mitochondrial transcription in the intestine of treated larvae with respect to DMSO-treated controls (Fig. 8A,B). Moreover, the treatment of larvae with AG490 caused a significant decrease in the number of intestinal proliferating cells (revealed by IHC with anti-pH3 antibody; Fig. 8C,D) and resulted in flattening of the intestinal mucosa (Fig. 8E,F). Taken together, these experiments demonstrate *in vivo* that Jak activity is required in zebrafish for normal mitochondrial transcription and its downstream effect on proliferation of the developing TeO and intestine.

### The zebrafish *stat3^−/−^* null mutant displays impairment of mitochondrial transcription and cell proliferation in CNS and intestine

**Fig. 9.**
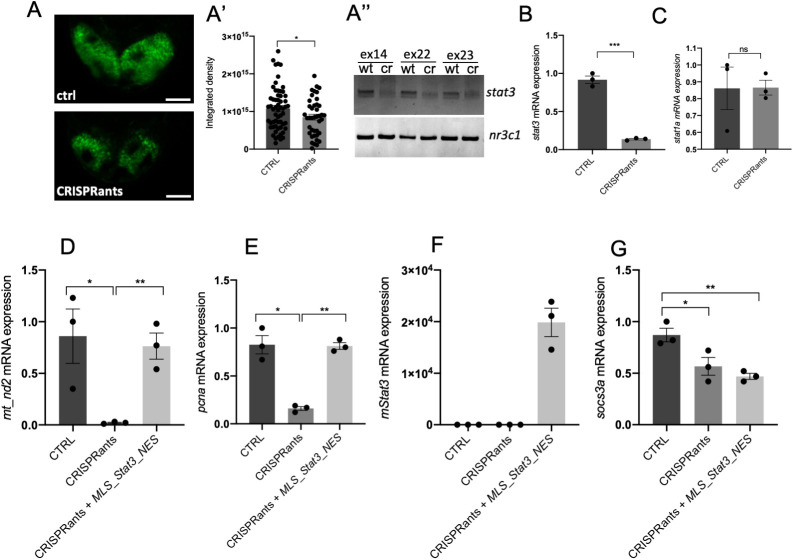
***stat3* CRISPRants show reduced mitochondrial transcription that is rescued by mitochondrial *Stat3*.** (A-A″) Representative images of 48 hpf *Tg(7xStat3-Hsv.Ul23:EGFP)^ia28^* transgenic zebrafish larvae (A). Fluorescent quantification of TeO of control and CRISPRant zebrafish larvae (A′). PCR amplification of the *stat3* gene on DNA extracts from control (wt) and CRISPRant (cr) larvae (*nr3c1* gene is used as an internal control) (A″). (B) qRT-PCR against *stat3* in 48 hpf control larvae and CRISPRants. (C) qRT-PCR against *stat1a* in 48 hpf control larvae and CRISPRants. (D) qRT-PCR against *mt_nd2* in 48 hpf control, CRISPRants and CRISPRants+*MLS_mStat3_NES* mRNA zebrafish larvae. (E) qRT-PCR against *pcna* in 48 hpf control, CRISPRants and CRISPRants+*MLS_mStat3_NES* mRNA zebrafish larvae. (F) qRT-PCR against *Stat3* in 48 hpf control, CRISPRants and CRISPRants+*MLS_mStat3_NES* mRNA zebrafish larvae. (G) qRT-PCR against *socs3a* in 48 hpf control, CRISPRants and CRISPRants+*MLS_mStat3_NES* mRNA zebrafish larvae. actb1 was used as an internal control. Data are mean±s.e.m. *P<0.05; **P<0.01; ***P<0.001 (unpaired two-tailed *t*-test on three independent biological samples, where *n* not specified)*.* ns, not significant. Scale bar: 50 μm.

Our results, obtained with kinase inhibitors and overexpression of *Stat3* constructs, indicate that STAT3 regulates mitochondrial transcription and cell proliferation. We decided to genetically support our observations. First, we used the zebrafish *stat3^ia23^* mutant (from now on called *stat3^−/−^*) (Peron et al., 2020), which is predicted to encode a premature stop codon at amino acid 456, thus lacking all functional domains including the dimerisation domain and the transactivation domain, harbouring Y705 and S727 phosphorylation sites, respectively. As reported in Peron et al. (2020), these mutants die within 1 month of age and they can be obtained only after breeding of adult *stat3^+/−^* zebrafish. As reported in Fig. S6B, no significant differences in *mt_nd2* transcript levels were detected on *stat3^+/+^*, *stat3^+/−^* and *stat3^−/−^* larvae analysed at 48 hpf. This result is probably due to genetic compensation, a process that frequently happens in zebrafish mutant lines (El-Brolosy et al., 2019), or to a maternal carryover of Stat3 protein. To overcome this issue and to determine whether genetic ablation of *stat3* alters mitochondrial transcription and cell proliferation in 48 hpf larvae, we analysed *stat3* ‘CRISPRants’ generated after injection of Cas9 protein and sgRNAs targeting the antisense strand of exons 14, 22 and 23 of the *stat3* gene: this approach is employed to target a gene and silence its RNA transcription, according to the principles of classic CRISPR/Cas9 (Strutt et al., 2018). We evaluated the efficiency of *stat3* targeting by injection of the *Tg(7xStat3-Hsv.Ul23:EGFP)^ia28^* reporter line characterised in Peron et al. (2020). Results show that Stat3-dependent fluorescence of reporters is significantly dampened in CRISPRants when compared with control larvae (Fig. 9A,A′), and PCR analysis of the *stat3* gene locus confirmed that *stat3* had been successfully mutagenised in exons 14, 22 and 23 (Fig. 9A″). Notably, qRT-PCR analysis of *stat3* in CRISPRants displayed a significant downregulation (Fig. 9B) and *stat1a*, which is upregulated in *stat3* knock-out mutants (Peron et al., 2020), was not significantly different in CRISPRants compared with controls, suggesting – as also revealed in Savage et al. (2019) and Buglo et al. (2020) – that compensation mechanisms are not triggered in CRISPRants (Fig. 9C). Hence, we decided to use CRISPRants for the analysis of *mt_nd2* and *pcna* expression levels. As reported in Fig. 9D,E, both transcripts were significantly downregulated in *stat3* CRISPRants, confirming again that Stat3 is involved in mitochondrial transcription and cell proliferation. Interestingly, injection of *MLS_mStat3_NES* mRNA in CRISPRants, although rescuing completely *mt_nd2* and *pcna* transcript levels, did not restore the nuclear activities of Stat3 (Fig. 9D-G).

**Fig. 10.**
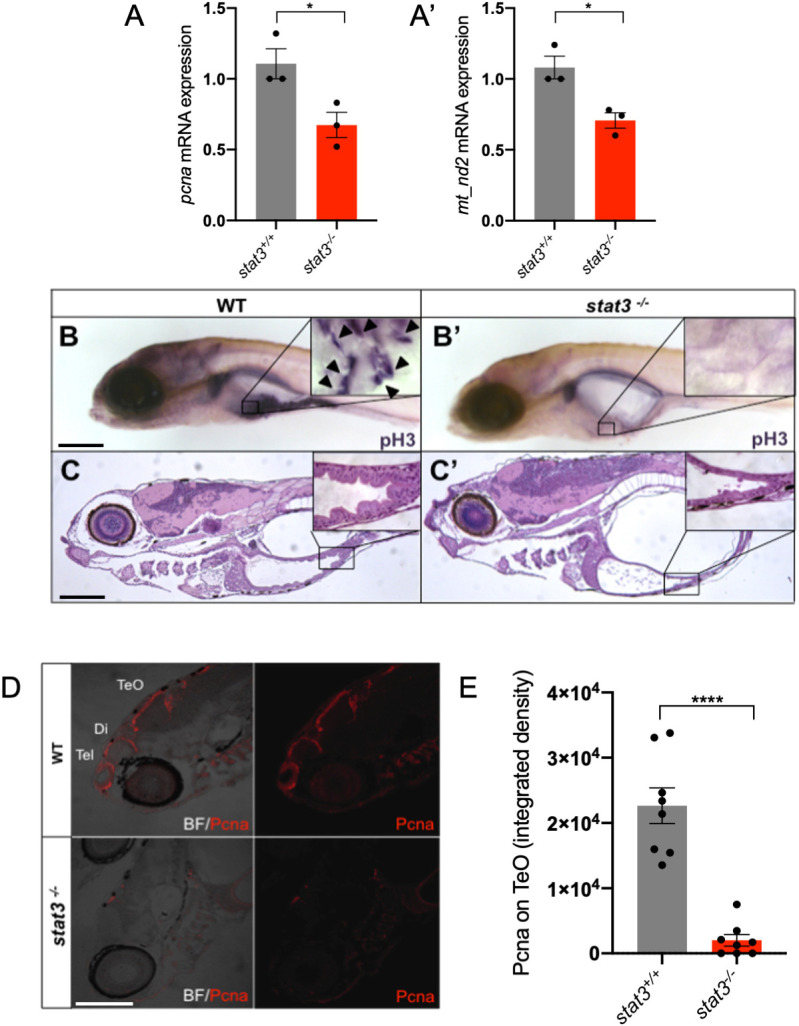
***stat3* knockout impairs normal mitochondrial transcription and cell proliferation in the intestine and brain of 6** **dpf zebrafish larvae.** (A,A′) Relative mRNA expression of *pcna* (A) and *mt_nd2* (A′) transcripts assayed by qRT-PCR in homogenised *stat3^−/−^* and WT siblings at 6 dpf; *zgapdh* was used as internal control (*P*-values=0.0358; 0.0182). (B,B′) Phospho-Histone-H3 (pH3) immunostaining of WT (B) and *stat3^−/−^* (B′) siblings at 6 dpf; inset shows magnification of boxed area of intestine. Arrowheads indicate pH3-positive cells. (C,C′) Hematoxylin and Eosin (H&E) staining on WT (C) and *stat3^−/−^* (C′) mutant sections at 6 dpf shows the complete loss of folding in the mutant intestinal epithelium; inset shows magnification of boxed area of intestine. (D) IF with anti-PCNA antibody on 6 dpf *stat3*^−/−^ mutants showing decrease of fluorescence in the CNS (BF, bright field; Di, diencephalon; Tel, telencephalon; TeO, tectum opticum). (E) Fluorescence quantification of Pcna protein on lateral sections of 6 dpf *stat3*^−/−^ mutants and WT siblings (*n*=8). Data are mean±s.e.m. *P<0.05; ****P<0.0001 (unpaired two-tailed *t*-test on three independent biological samples, where *n* not specified)*.* Scale bars: 200 μm.

In agreement with our previous results, when analysed at 6 dpf *stat3* knockout displayed a significant and clear reduction of both *mt_nd2* and *pcna* transcripts (Fig. 10A,A′), endorsing the link between Stat3 mitochondrial functions and its role in the regulation of cell proliferation. Interestingly, no overt structural alteration was present in intestinal or brain mitochondria of 6 dpf *stat3^−/−^* larvae analysed by TEM (Fig. S7A). Moreover, in order to evaluate the volume of mitochondria, we crossed *stat3* mutants with the *Tg(CoxVIII-mls:EGFP)* transgenic line that expresses a mitochondria-localised form of enhanced GFP. No clear change in the total mitochondria volume was present in the intestine of *stat3^−/−^* larvae compared with *stat3^+/+^* sibling larvae (Fig. S7B,C). Together with previous results, this highlights that mitoSTAT3 is only acting as a regulator of mitochondrial transcription, without impacting on mitochondria biogenesis or homeostasis. Notably, ∼70% of *stat3^−/−^* larvae displayed severe defects in the development of intestinal epithelium (Peron et al., 2020): as revealed by pH3 immunostaining, intestinal mitoses were almost absent (Fig. 10B,B′) and the intestine failed to fold (Fig. 10C,C′). These phenotypic alterations were almost identical to those induced by AG490 treatment (Fig. 8B,C). At 6 dpf, *stat3^−/−^* larvae also showed impaired CNS cell proliferation in the telencephalon, the diencephalon and the TeO, in which Pcna was found to be reduced down to 15% compared with *stat3^+/+^* siblings, supporting, once again, the requirement of Stat3 for the maintenance of normal proliferation in the brain (Fig. 10D,E).

## DISCUSSION

Using the zebrafish model and taking advantage of a STAT3 harbouring both an MLS and an NES, we explored how mitoSTAT3 may act inside the mitochondrion. Interestingly, as mitochondrial mRNAs are reduced in *stat3^ia23/ia23^* zebrafish null mutants and CRISPRants, are decreased in embryos treated with the Jak2 kinase inhibitor AG490, and the effect of *MLS_Stat3_NES* in promoting mitochondrial gene expression is abolished by Balapiravir (a mitochondrial RNA polymerase inhibitor), our data strongly support, *in vivo*, a direct link between mitoSTAT3 activity and mitochondrial transcription. This is consistent with the mitochondrial transcriptional role of mitoSTAT3 found *in vitro* in murine ESCs, as previously reported (Carbognin et al., 2016). On the other hand, quite surprisingly for a transcription factor, *MLS_Stat3_NES* mutated in its DNA-binding domain is still able to increase mitochondrial transcription. This result suggests that STAT3, differently from what is hypothesised in Macias et al. (2014), does not regulate mtDNA transcription by binding STAT3-responsive elements located in the mtDNA, with the differences between the eukaryotic and the prokaryotic transcriptional machineries operating in the nucleus and mitochondria, respectively. We have previously reported that Stat3 binds mtDNA in ESCs (Carbognin et al., 2016), therefore we hypothesise that such binding is mediated by additional proteins, the identification of which will be the aim of future studies.

One of the most fascinating aspects of mitochondria evolution is their progressive incorporation in the machinery of cell regulatory activities such as cell proliferation and apoptosis (Antico Arciuch et al., 2012). By showing that mitoSTAT3-driven mitochondrial transcription controls cell proliferation, at least in intestinal and tectal undifferentiated progenitor cells, our data partially answer the open questions about the mechanisms that synchronise mitochondrial and nuclear activities during cell proliferation.

Canonical STAT3 activation depends on different modifications, such as the phosphorylation at tyrosine 705 (Y705) that induces dimerisation and translocation to the nucleus, and at serine 727 (S727), the function of which has been reported to have unclear effects on STAT3 nuclear transcriptional activity (Decker and Kovarik, 2000; Huang et al., 2014; Tesoriere et al., 2021). On the other hand, the post-translational modifications required for mitoSTAT3 import and activity in mitochondria have not been clearly dissected so far, although phosphorylation at S727 has been found to both activate OXPHOS complexes I and II, and suppress ROS production and cytochrome c release following ischemic injury (Meier and Larner, 2014). More recently, STAT3 phosphorylation at S727 was also found to be required for STAT3-mediated regulation of ER Ca^2+^ fluxes and apoptosis through the regulation of the mitochondrial Ca^2+^ uptake (Avalle et al., 2019). Here, we provide *in vivo* evidence, confirmed in ESCs, that phosphorylation of STAT3 Y705, although being required for precise mitochondrial import of STAT3, is unable to induce mitochondrial gene expression. On the other hand, in accordance with the results obtained by Wegrzyn et al. (2009), we show that mitochondrial STAT3 transcriptional activity *in vivo* is totally dependent on phosphorylation of the ERK target S727. Experiments in mouse ESCs allowed us to clearly demonstrate that phosphorylated S727 is not required for mitochondrial localisation of STAT3 but, rather, for its activity once imported into the organelle. *In vivo* experiments performed in zebrafish larvae show that both mitoSTAT3-mediated mtDNA transcription and cell proliferation are repressed by targeting S727 with a MEK inhibitor. In order to test more directly the involvement of MEK1,2 in S727 phosphorylation we used a more specific MEK1,2 inhibitor: PD0325901 (Fig. S5D). Surprisingly, this inhibitor does not have any effect on mitochondrial transcription. It is known from literature that other kinases, such as CDK8 (Martinez-Fabregas et al., 2020), TBK-1 (Balic et al., 2020) and FAK cells (Visavadiya et al., 2016) are able to target S727. Therefore, it is likely that PD98059 is indeed targeting S727 through MEKs other than MEK1,2 or that, at least in zebrafish, its specificity is broader than expected.

By dissecting the roles of Y705 and S727 phosphorylation in the mitochondrial-specific activity of STAT3 both in zebrafish larvae and mouse ESCs, our results add further insight into the specificity of mitochondrial STAT3 in the regulation of cellular processes, previously thought to be dependent exclusively on canonical (nuclear) STAT3. Together with the fact that mitochondrial STAT3 has been identified as a contributor to RAS-dependent cellular transformation (Gough et al., 2009), we support the idea that ERK-mitoSTAT3-mediated mitochondrial transcription might be a key process in cancer development, especially in the intestine, where we demonstrate here and in Peron et al. (2020) that cell proliferation is STAT3-dependent. Considering that, to date, the vast majority of STAT3-targeted cancer therapeutic approaches focus only on its canonical functions, our findings imply mitochondrial STAT3-specific transcriptional activity as a significant molecular mechanism to be targeted.

## MATERIALS AND METHODS

### Animal husbandry and lines

Zebrafish were staged and fed as described by Kimmel et al. (1995) and maintained in large-scale aquaria systems. Embryos were obtained by natural mating, raised at 28°C in Petri dishes containing fish water (50X: 25 g Instant Ocean, 39.25 g CaSO_4_ and 5 g NaHCO_3_ for 1 l) and kept in a 12 h light/12 h dark (LD) cycle. All experimental procedures complied with European Legislation for the Protection of Animals used for Scientific Purposes (Directive 2010/63/EU) and were approved by the Animal Experiments Committee of the University of Padua and the Italian Ministry of Health (23-2015-PR).

*stat3^ia23^* mutants and *Tg(7xStat3-Hsv.Ul23:EGFP)^ia28^* transgenic zebrafish are described in Peron et al. (2020). The *Tg(CoxVIII-mls:EGFP)* transgenic zebrafish line is described in Martorano et al. (2019).

### Drug treatments

The following chemical compounds were used: AG490 (T3434, Sigma-Aldrich); PD98059 (PHZ1164, Thermo Fisher Scientific); PD0325901 (Axon 1386, Axon Medchem); and Balapiravir (HY-10443, DBA). Before drug administration, a hole was made in the chorion of 8 hpf embryos, whereas 24 hpf embryos were dechorionated. All drugs were dissolved in DMSO and stored in small aliquots at −20°C. We performed 100 µM AG490 treatment from 24 to 48 hpf or from 24 to 72 hpf. We administered 60 µM AG490 in 3-6 dpf treatments, and 12.5 µM PD98059 or 10 μM PD325901 (same treatment as PD98059) treatments were administered from 24 to 48 hpf. Balapiravir solution (50 µM) was administered from 8 to 48 hpf. After treatments, embryos were anaesthetised and fixed in either 4% paraformaldehyde (PFA) (158127, Sigma-Aldrich) in PBS for ISH, FISH and IHC or in TRI Reagent^®^ (T9424, Sigma-Aldrich) for qRT-PCR analysis.

### CRISPRant generation

sgRNA against exon 14 of the *stat3* gene was produced as described in Peron et al. (2020). sgRNAs against exon 22 and 23 of the *stat3* gene were designed with CHOPCHOP software https://chopchop.cbu.uib.no/ and provided by Synthego.

Cas9 protein (M0646, New England Biolabs) and sgRNAs against antisense of exons 14, 22 and 23 of *stat3* genes were injected into one-cell-stage eggs. Subsequently, 48 hpf injected larvae were collected for DNA and RNA extraction and for imaging. Sequences of sgRNAs and of primers used for genotyping are listed in Tables S1 and S2, respectively.

### mRNAs synthesis and injection

*mStat3*, *mStat3_Y705F* and *mStat3_S727A* coding sequences were obtained from pCEP4-*Stat3-WT*, pCEP4-*Stat3-Y705F* and pCEP4-*Stat3-S727A* plasmids (a kind gift of the Poli Lab; Department of Molecular Biotechnology and Health Sciences, Molecular Biotechnology Center, University of Turin, Italy) and sub-cloned into a pCS2+ backbone using the In-Fusion^®^ HD Cloning Kit (Clontech). *MLS_mStat3_NES* CDS, containing the murine *Stat3* cDNA flanked by an MLS and an NES, was subcloned into a pCS2+ plasmid from a 70_pPB-CAG+MLS+*Stat3*+NES-pA-pgk-hph-2-2 plasmid by digestion with *XbaI* and *BamHI*. Mutated forms of *MLS_mStat3_NES* mRNA were obtained from pCS2+*MLS_mStat3_NES* by site-directed mutagenesis using the Q5^®^ Site-Directed Mutagenesis Kit (New England Biolabs); primers are indicated in Table S3.

mRNAs were *in vitro* transcribed using the mMESSAGE mMACHINE^®^ SP6 Transcription Kit (Thermo Fisher Scientific) and purified using the RNA Clean and Concentrator kit (Zymo Research). A mix containing mRNA (30 ng/µl for *Stat3-WT*, *Stat3-Y705F*, *Stat3-S727A*; 50 ng/µl for *MLS_Stat3_NES*), Danieau injection Buffer and Phenol Red injection dye, was injected into one-cell-stage embryos.

### mRNA isolation and qRT-PCR

For expression analysis, total RNA was extracted from pools of 15 7 dpf larvae or 35 48 hpf embryos with TRIzol reagent (15596018, Thermo Fisher Scientific). mRNA was treated with RQ1 RNase-Free DNase (M6101, Promega) and then used for cDNA synthesis with Superscript III Reverse Transcriptase (18080-044, Invitrogen) according to the manufacturer's protocol. qPCR was performed in triplicate with EvaGreen method using a Rotor-gene Q (Qiagen) and the 5× HOT FIREPol^®^ EvaGreen^®^ qPCR Mix Plus (08-36-00001, Solis BioDyne) following the manufacturer's protocol. The cycling parameters were: 95°C for 14 min, followed by 45 cycles at 95°C for 15 s, 60°C for 35 s and 72°C for 25 s. Threshold cycles (Ct) and dissociation curves were generated automatically by Rotor-Gene Q series software. Sequences of specific primers used in this work for qRT-PCR and RT-PCR are listed in Table S4. Primers were designed using the software Primer 3 (https://primer3.ut.ee/). Sample Ct values were normalised with Ct values from zebrafish *gapdh* and results were obtained following the method described in Livak and Schmittgen (2001).

### Immunoblotting and mitochondria isolation

Immunoblotting was performed as previously described in Carbognin et al. (2016). The following antibodies were used: anti-STAT3 mouse monoclonal (9139, Cell Signaling Technology, 1:1000), anti-GAPDH mouse monoclonal (MAB374, Millipore, 1:1000), anti-VDAC1 rabbit polyclonal (ab15895, Abcam, 1:1000), anti-Lamin (sc-6217, Santa Cruz Biotechnology, 1:1000), anti-βActin mouse monoclonal (MA1-744, Invitrogen, 1:10,000) and anti-pSTAT3 S727 rabbit monoclonal (9134, Cell Signaling Technology). Mitochondria from mouse ESCs were isolated using Mitochondria Isolation Kit (89874, Thermo Fisher Scientific).

### 3,3′-Diaminobenzidine staining

Cells were fixed in a 24-well plate with 4% PFA in PBS (pH 7.4) for 30 min at room temperature (RT). After fixation cells were washed five times with PBS (5 min each), blocked and permeabilised with 5% normal goat serum and 0.1% saponin in PBS for 30 min, and then incubated with primary antibody anti-STAT3 mouse monoclonal (9139, Cell Signaling Technology) overnight at 4°C in PBS 5% normal goat serum and 0.05% saponin. After five washes with PBS (5 min each), cells were incubated with HRP-conjugated Fab fragments of the secondary antibody for 2 h at RT. After five washes, cells were incubated in the DAB solution (0.01 g DAB in 20 ml TRIS-HCl buffer plus 30% H_2_O_2_ solution added immediately before use). The samples were then postfixed with 1% osmium tetroxide plus potassium ferrocyanide 1% in 0.1 M sodium cacodylate buffer for 1 h at 4°C. After three water washes, samples were dehydrated in a graded ethanol series and embedded in an epoxy resin (Sigma-Aldrich). Ultrathin sections (60-70 nm) were obtained with an Ultrotome V (LKB) ultramicrotome, counterstained with uranyl acetate and lead citrate and viewed with a Tecnai G2 (FEI) transmission electron microscope operating at 100 kV. Images were captured with a Veleta (Olympus Soft Imaging System) digital camera.

### Immunofluorescence

ESCs were grown and transfected as previously described by Carbognin et al. (2016). For IF, ESCs were fixed for 10 min in cold methanol at −20°C, washed in TBS, permeabilised for 10 min with TBST (Tris-buffered saline/Tween 20)+0.3% Triton X-100 at RT, and blocked for 45 min in TBS+3% goat serum at RT. The cells were incubated overnight at 4°C with primary antibodies anti-STAT3 mouse monoclonal (9139, Cell Signaling Technology, 1:100) and anti-ATAD3A rabbit monoclonal (224485, AB-Biotechnologies, 1:100). After washing with TBS, the cells were incubated with secondary antibodies (Alexa Fluor 568, raised in donkey anti-mouse IgG, diluted 1:500, Thermo Fisher, A-11036; Alexa Fluor 488, raised in goat anti-rabbit IgG, diluted 1:500, Thermo Fisher, A-21206) for 30 min at RT. Cells were mounted with ProLong^®^ Gold Antifade Mountant with DAPI (P36941, Life Technologies) or HOECHST 33342 (62249, Thermo Fisher Scientific) where specified. Images were acquired using a Leica SP2 confocal microscope equipped with a CCD camera.

### *In situ* hybridisation

Whole mount RNA *in situ* hybridisation (WISH) on zebrafish embryos was performed as previously described (Thisse et al., 1993). It is worth mentioning that treated and control embryos were hybridised together. The *stat3* probe was obtained by PCR amplification from embryo cDNA using *stat3*_probe-fw (5′-TGCCACCAACATCCTAGTGT-3′) and *stat3*_probe-rv (5′-GCTTGTTTGCACTTTTGACTGA-3′) primers. The *mt_nd2* probe was obtained by PCR amplification from embryo cDNA using *mt_nd2*-fw (5′-GCAGTAGAAGCCACCACAAA-3′) and *mt_nd2*-rv (5′-GGAATGCCGCGGATGTTATA-3′) primers. The *pcna* probe was obtained as described by Baumgart et al. (2014). The *sox9b* probe was obtained as described by Chiang et al. (2001). FISH was performed with FastBlue or TSA-amplification kit (Invitrogen) as described by Lauter et al. (2011).

### Combined IHC-WISH

Combined IHC and WISH were carried out as follows. IHC was performed as described in Giuliodori et al. (2018), using anti-PCNA mouse monoclonal (ab29, Abcam, 1:500) as primary antibody, and goat anti-mouse (Alexa Fluor 488, raised in donkey anti-mouse IgG, diluted 1:500, Thermo Fisher, A32766) as secondary antibody, to fluorescently detect Pcna-positive cells.

After IHC, samples were rinsed in 1× PBS and subjected to WISH, according to Thisse and Thisse (2008). The following digoxigenin-labelled riboprobes were used: *pcna*, *stat3* and *mt_nd2*. Alkaline phosphatase-mediated Fast Blue staining was performed by incubating samples in 0.25 mg/ml Fast Blue BB (F3378, Sigma-Aldrich), 0.25 mg/ml Naphtol-AS-MX-phosphate (N5000, Sigma-Aldrich), 100 mM Tris HCl (pH 8.2), 50 mM MgCl_2_, 100 mM NaCl and 0.1% Tween 20. After glycerol-based mounting, green (from IHC) and far-red (from WISH) emissions were acquired using a Leica SP5 confocal system. Signals were analysed by Volocity 6.0 software (Perkin Elmer) and images organised by Adobe Photoshop v. 21.1.1.

### Transmission electron microscopy analysis

Larvae were anaesthetised and fixed with 2.5% glutaraldehyde in 0.1 M sodium cacodylate buffer. After that, samples were dehydrated, embedded in epoxy resin, and prepared according to standard protocols by the Trasmission Electron Microscopy facility at the Department of Biology, University of Padova (Italy).

### Statistical analysis

Statistical analysis was performed with GraphPad Prism V6.0. Data are presented as mean±s.e.m. and statistical analysis was determined using the unpaired two-tailed Student's *t*-test. The *P*-values are indicated with the following symbols: *P<0.05; **P<0.01; ***P<0.001; ****P<0.0001. For quantitative analysis, the sample size for each experiment was calculated assuming a confidence level of 95% (*z*-score 1.96), a standard deviation of 0.5 and a confidence interval (margin of error) of 5%.

## Supplementary Material

Supplementary information

Reviewer comments

## References

[DEV199477C1] Alessi, D. R., Cuenda, A., Cohen, P., Dudley, D. T. and Saltiel, A. R. (1995). PD 098059 is a specific inhibitor of the activation of mitogen-activated protein kinase kinase in vitro and in vivo. *J. Biol. Chem.* 270, 27489-27494. 10.1074/jbc.270.46.27489 7499206

[DEV199477C2] Antico Arciuch, V. G., Elguero, M. E., Poderoso, J. J. and Carreras, M. C. (2012). Mitochondrial regulation of cell cycle and proliferation. *Antioxid. Redox Signal.* 16, 1150-1180. 10.1089/ars.2011.4085 21967640 PMC3315176

[DEV199477C3] Avalle, L., Camporeale, A., Morciano, G., Caroccia, N., Ghetti, E., Orecchia, V., Viavattene, D., Giorgi, C., Pinton, P. and Poli, V. (2019). STAT3 localizes to the ER, acting as a gatekeeper for ER-mitochondrion Ca^2+^ fluxes and apoptotic responses. *Cell Death Differ.* 26, 932-942. 10.1038/s41418-018-0171-y 30042492 PMC6214529

[DEV199477C4] Balic, J. J., Albargy, H., Luu, K., Kirby, F. J., Jayasekara, W. S. N., Mansell, F., Garama, D. J., De Nardo, D., Baschuk, N., Louis, C. et al. (2020). STAT3 serine phosphorylation is required for TLR4 metabolic reprogramming and IL-1β expression. *Nat. Commun.* 11, 3816. 10.1038/s41467-020-17669-5 32732870 PMC7393113

[DEV199477C5] Baumgart, M., Groth, M., Priebe, S., Savino, A., Testa, G., Dix, A., Ripa, R., Spallotta, F., Gaetano, C., Ori, M. et al. (2014). RNA-seq of the aging brain in the short-lived fish *N. furzeri* – conserved pathways and novel genes associated with neurogenesis. *Aging Cell* 13, 965-974. 10.1111/acel.12257 25059688 PMC4326923

[DEV199477C6] Becker, S., Groner, B. and Müller, C. W. (1998). Three-dimensional structure of the Stat3β homodimer bound to DNA*.* *Nature* 394, 145-151 10.1038/28101 9671298

[DEV199477C7] Buglo, E., Sarmiento, E., Martuscelli, N. B., Sant, D. W., Danzi, M. C., Abrams, A. J., Dallman, J. E. and Züchner, S. (2020). Genetic compensation in a stable slc25a46 mutant zebrafish: a case for using F0 CRISPR mutagenesis to study phenotypes caused by inherited disease. *PLoS ONE* 15, e0230566. 10.1371/journal.pone.0230566 32208444 PMC7092968

[DEV199477C8] Burdon, T., Chambers, I., Stracey, C., Niwa, H. and Smith, A. (1999). Signaling mechanisms regulating self-renewal and differentiation of pluripotent embryonic stem cells. *Cells Tissues Organs* 165, 131-143. 10.1159/000016693 10592385

[DEV199477C9] Carbognin, E., Betto, R. M., Soriano, M. E., Smith, A. G. and Martello, G. (2016). Stat3 promotes mitochondrial transcription and oxidative respiration during maintenance and induction of naive pluripotency. *EMBO J.* 35, 618-634. 10.15252/embj.201592629 26903601 PMC4801951

[DEV199477C10] Chiang, E. F.-L., Pai, C.-I., Wyatt, M., Yan, Y.-L., Postlethwait, J. and Chung, B.-C. (2001). Two sox9 genes on duplicated zebrafish chromosomes: Expression of similar transcription activators in distinct sites. *Dev. Biol.* 231, 149-163. 10.1006/dbio.2000.0129 11180959

[DEV199477C11] Decker, T. and Kovarik, P. (2000). Serine phosphorylation of STATs. *Oncogene* 19, 2628-2637. 10.1038/sj.onc.1203481 10851062

[DEV199477C12] El-Brolosy, M. A., Kontarakis, Z., Rossi, A., Kuenne, C., Günther, S., Fukuda, N., Kikhi, K., Boezio, G. L. M., Takacs, C. M., Lai, S.-L. et al. (2019). Genetic compensation triggered by mutant mRNA degradation. *Nature* 568, 193-197. 10.1038/s41586-019-1064-z 30944477 PMC6707827

[DEV199477C13] Facchinello, N., Skobo, T., Meneghetti, G., Colletti, E., Dinarello, A., Tiso, N., Costa, R., Gioacchini, G., Carnevali, O., Argenton, F. et al. (2017). *nr3c1* null mutant zebrafish are viable and reveal DNA-binding-independent activities of the glucocorticoid receptor. *Sci. Rep.* 7, 4371. 10.1038/s41598-017-04535-6 28663543 PMC5491532

[DEV199477C14] Feng, J. Y., Xu, Y., Barauskas, O., Perry, J. K., Ahmadyar, S., Stepan, G., Yu, H., Babusis, D., Park, Y., McCutcheon, K. et al. (2016). Role of mitochondrial RNA polymerase in the toxicity of nucleotide inhibitors of hepatitis C virus. *Antimicrob. Agents Chemother.* 60, 806-817. 10.1128/AAC.01922-15 26596942 PMC4750701

[DEV199477C15] Fouse, S. D. and Costello, J. F. (2013). Cancer stem cells activate STAT3 the EZ way. *Cancer Cell* 23, 711-713. 10.1016/j.ccr.2013.05.016 23763996 PMC3706087

[DEV199477C16] Galant, S., Furlan, G., Coolen, M., Dirian, L., Foucher, I. and Bally-Cuif, L. (2016). Embryonic origin and lineage hierarchies of the neural progenitor subtypes building the zebrafish adult midbrain. *Dev. Biol.* 420, 120-135. 10.1016/j.ydbio.2016.09.022 27693369 PMC5156517

[DEV199477C17] Ghoshal, S., Fuchs, B. C. and Tanabe, K. K. (2016). STAT3 is a key transcriptional regulator of cancer stem cell marker CD133 in HCC. *Hepatobiliary Surg. Nutr.* 5, 201-203. 10.21037/hbsn.2016.03.02 27275460 PMC4876240

[DEV199477C18] Giuliodori, A., Beffagna, G., Marchetto, G., Fornetto, C., Vanzi, F., Toppo, S., Facchinello, N., Santimaria, M., Vettori, A., Rizzo, S. et al. (2018). Loss of cardiac Wnt/β-catenin signalling in desmoplakin-deficient AC8 zebrafish models is rescuable by genetic and pharmacological intervention. *Cardiovasc. Res.* 114, 1082-1097. 10.1093/cvr/cvy057 29522173

[DEV199477C19] Gough, D. J., Corlett, A., Schlessinger, K., Wegrzyn, J., Larner, A. C. and Levy, D. E. (2009). Mitochondrial STAT3 supports Ras-dependent oncogenic transformation. *Science* 324, 1713-1716. 10.1126/science.1171721 19556508 PMC2840701

[DEV199477C20] Gough, D. J., Koetz, L. and Levy, D. E. (2013). The MEK-ERK pathway is necessary for serine phosphorylation of mitochondrial STAT3 and ras-mediated transformation. *PLoS ONE* 8, e83395. 10.1371/journal.pone.0083395 24312439 PMC3843736

[DEV199477C21] Grivennikov, S., Karin, E., Terzic, J., Mucida, D., Yu, G.-Y., Vallabhapurapu, S., Scheller, J., Rose-John, S., Cheroutre, H., Eckmann, L. et al. (2009). IL-6 and Stat3 are required for survival of intestinal epithelial cells and development of colitis-associated cancer. *Cancer Cell* 15, 103-113. 10.1016/j.ccr.2009.01.001 19185845 PMC2667107

[DEV199477C22] Gurbuz, V., Konac, E., Varol, N., Yilmaz, A., Gurocak, S., Menevse, S. and Sozen, S. (2014). Effects of AG490 and S3I-201 on regulation of the JAK/STAT3 signaling pathway in relation to angiogenesis in TRAIL-resistant prostate cancer cells in vitro. *Oncol. Lett.* 7, 755-763. 10.3892/ol.2014.1795 24520293 PMC3919920

[DEV199477C23] Horvath, C. M., Wen, Z. and Darnell, J. E. Jr. (1995). A STAT protein domain that determines DNA sequence recognition suggests a novel DNA-binding domain. *Genes Dev.* 9, 984-994. 10.1101/gad.9.8.984 7774815

[DEV199477C24] Huang, G., Yan, H., Ye, S., Tong, C. and Ying, Q.-L. (2014). STAT3 phosphorylation at tyrosine 705 and serine 727 differentially regulates mouse ESC fates. *Stem Cells* 32, 1149-1160. 10.1002/stem.1609 24302476 PMC4181708

[DEV199477C25] Johnston, P. A. and Grandis, J. R. (2011). STAT3 signaling: anticancer strategies and challenges. *Mol. Interv.* 11, 18-26. 10.1124/mi.11.1.4 21441118 PMC3063716

[DEV199477C26] Kim, J.-H., Yoon, M.-S. and Chen, J. (2009). Signal transducer and activator of transcription 3 (STAT3) mediates amino acid inhibition of insulin signaling through serine 727 phosphorylation. *J. Biol. Chem.* 284, 35425-35432. 10.1074/jbc.M109.051516 19875458 PMC2790971

[DEV199477C27] Kimmel, C. B., Ballard, W. W., Kimmel, S. R., Ullmann, B. and Schilling, T. F. (1995). Stages of embryonic development of the zebrafish. *Dev. Dyn.* 203, 253-310. 10.1002/aja.1002030302 8589427

[DEV199477C28] Lauter, G., Söll, I. and Hauptmann, G. (2011). Two-color fluorescent in situ hybridization in the embryonic zebrafish brain using differential detection systems. *BMC Dev. Biol.* 11, 43. 10.1186/1471-213X-11-43 21726453 PMC3141750

[DEV199477C29] Liang, J., Wang, D., Renaud, G., Wolfsberg, T. G., Wilson, A. F. and Burgess, S. M. (2012). The stat3/socs3a pathway is a key regulator of hair cell regeneration in zebrafish stat3/socs3a pathway: regulator of hair cell regeneration. *J. Neurosci.* 32, 10662-10673. 10.1523/JNEUROSCI.5785-10.2012 22855815 PMC3427933

[DEV199477C30] Liu, Y., Sepich, D. S. and Solnica-Krezel, L. (2017). Stat3/Cdc25a-dependent cell proliferation promotes embryonic axis extension during zebrafish gastrulation. *PLoS Genet.* 13, e1006564. 10.1371/journal.pgen.1006564 28222105 PMC5319674

[DEV199477C31] Livak, K. J. and Schmittgen, T. D. (2001). Analysis of relative gene expression data using real-time quantitative PCR and the 2^−ΔΔCT^ method. *Methods* 25, 402-408. 10.1006/meth.2001.1262 11846609

[DEV199477C32] Macias, E., Rao, D., Carbajal, S., Kiguchi, K. and DiGiovanni, J. (2014). Stat3 binds to mtDNA and regulates mitochondrial gene expression in keratinocytes. *J. Investig. Dermatol.* 134, 1971-1980. 10.1038/jid.2014.68 24496235 PMC4057971

[DEV199477C33] Mantel, C., Messina-Graham, S., Moh, A., Cooper, S., Hangoc, G., Fu, X.-Y. and Broxmeyer, H. E. (2012). Mouse hematopoietic cell-targeted STAT3 deletion: stem/progenitor cell defects, mitochondrial dysfunction, ROS overproduction, and a rapid aging-like phenotype. *Blood* 120, 2589-2599. 10.1182/blood-2012-01-404004 22665934 PMC3460681

[DEV199477C34] Martello, G., Bertone, P. and Smith, A. (2013). Identification of the missing pluripotency mediator downstream of leukaemia inhibitory factor. *EMBO J.* 32, 2561-2574. 10.1038/emboj.2013.177 23942233 PMC3791366

[DEV199477C35] Martinez-Fabregas, J., Wang, L., Pohler, E., Cozzani, A., Wilmes, S., Kazemian, M., Mitra, S. and Moraga, I. (2020). CDK8 Fine-Tunes IL-6 transcriptional activities by limiting STAT3 resident time at the gene loci. *Cell Rep.* 33, 108545-108560. 10.1016/j.celrep.2020.108545 33357429 PMC7773550

[DEV199477C36] Martorano, L., Peron, M., Laquatra, C., Lidron, E., Facchinello, N., Meneghetti, G., Tiso, N., Rasola, A., Ghezzi, D. and Argenton, F. (2019). The zebrafish orthologue of the human hepatocerebral disease gene *MPV17* plays pleiotropic roles in mitochondria. *Dis Model Mech.* 12, dmm037226. 10.1242/dmm.037226 30833296 PMC6451431

[DEV199477C37] Matsuda, T., Nakamura, T., Nakao, K., Arai, T., Katsuki, M., Heike, T. and Yokota, T. (1999). STAT3 activation is sufficient to maintain an undifferentiated state of mouse embryonic stem cells. *EMBO J.* 18, 4261-4269. 10.1093/emboj/18.15.4261 10428964 PMC1171502

[DEV199477C38] Matthews, J. R., Sansom, O. J. and Clarke, A. R. (2011). Absolute requirement for STAT3 function in small-intestine crypt stem cell survival. *Cell Death Differ.* 18, 1934-1943. 10.1038/cdd.2011.77 21637293 PMC3214915

[DEV199477C39] Minami, M., Inoue, M., Wei, S., Takeda, K., Matsumoto, M., Kishimoto, T. and Akira, S. (1996). STAT3 activation is a critical step in gp130-mediated terminal differentiation and growth arrest of a myeloid cell line. *Proc. Natl. Acad. Sci. USA* 93, 3963-3966. 10.1073/pnas.93.9.3963 8632998 PMC39468

[DEV199477C40] Meier, J. A. and Larner, A. C. (2014). Toward a new STATe: the role of STATs in mitochondrial function. *Semin. Immunol.* 26, 20-28. 10.1016/j.smim.2013.12.005 24434063 PMC4321820

[DEV199477C41] Mohr, A., Fahrenkamp, D., Rinis, N. and Müller-Newen, G. (2013). Dominant-negative activity of the STAT3-Y705F mutant depends on the N-terminal domain. *Cell Commun. Signal.* 11, 83. 10.1186/1478-811X-11-83 24192293 PMC3833267

[DEV199477C42] Ni, C.-W., Hsieh, H.-J., Chao, Y.-J. and Wang, D. L. (2004). Interleukin-6-induced JAK2/STAT3 signaling pathway in endothelial cells is suppressed by hemodynamic flow. *Am. J. Physiol. Cell Physiol.* 287, C771-C780. 10.1152/ajpcell.00532.2003 15151905

[DEV199477C43] Oates, A. C., Wollberg, P., Pratt, S. J., Paw, B. H., Johnson, S. L., Ho, R. K., Postlethwait, J. H., Zon, L. I. and Wilks, A. F. (1999). Zebrafish stat3 is expressed in restricted tissues during embryogenesis and stat1 rescues cytokine signaling in a STAT1-deficient human cell line. *Dev. Dyn.* 215, 352-370. 10.1002/(SICI)1097-0177(199908)215:4<352::AID-AJA7>3.0.CO;2-J 10417824

[DEV199477C44] O'Shea, J. J., Schwartz, D. M., Villarino, A. V., Gadina, M., McInnes, I. B. and Laurence, A. (2015). The JAK-STAT pathway: impact on human disease and therapeutic intervention. *Annu. Rev. Med.* 66, 311-328. 10.1146/annurev-med-051113-024537 25587654 PMC5634336

[DEV199477C45] Park, J.-S., Lee, J., Lim, M.-A., Kim, E.-K., Kim, S.-M., Ryu, J.-G., Lee, J. H., Kwok, S.-K., Park, K.-S., Kim, H.-Y. et al. (2014). JAK2-STAT3 blockade by AG490 suppresses autoimmune arthritis in mice via reciprocal regulation of regulatory T Cells and Th17 cells. *J. Immunol.* 192, 4417-4424. 10.4049/jimmunol.1300514 24688026

[DEV199477C46] Peron, M., Dinarello, A., Meneghetti, G., Martorano, L., Facchinello, N., Vettori, A., Licciardello, G., Tiso, N. and Argenton, F. (2020). The stem-like STAT3-responsive cells of zebrafish intestine are WNT/β-catenin dependent. *Development* 147, dev188987. 10.1242/dev.188987 32467235 PMC7328161

[DEV199477C47] Qin, H. R., Kim, H.-J., Kim, J.-Y., Hurt, E. M., Klarmann, G. J., Kawasaki, B. T., Duhagon Serrat, M. A. and Farrar, W. L. (2008). Activation of signal transducer and activator of transcription 3 through a phosphomimetic serine 727 promotes prostate tumorigenesis independent of tyrosine 705 phosphorylation. *Cancer Res.* 68, 7736-7741. 10.1158/0008-5472.CAN-08-1125 18829527 PMC2859454

[DEV199477C48] Savage, A. M., Kurusamy, S., Chen, Y., Jiang, Z., Chhabria, K., MacDonald, R. B., Kim, H. R., Wilson, H. L., van Eeden, F. J. M., Armesilla, A. L. et al. (2019). tmem33 is essential for VEGF-mediated endothelial calcium oscillations and angiogenesis. *Nat. Commun.* 10, 732. 10.1038/s41467-019-08590-7 30760708 PMC6374405

[DEV199477C49] Shi, X., Zhang, H., Paddon, H., Lee, G., Cao, X. and Pelech, S. (2006). Phosphorylation of STAT3 serine-727 by cyclin-dependent kinase 1 is critical for nocodazole-induced mitotic arrest. *Biochemistry* 45, 5857-5867. 10.1021/bi052490j 16669628

[DEV199477C50] Smith, A. G., Heath, J. K., Donaldson, D. D., Wong, G. G., Moreau, J., Stahl, M. and Rogers, D. (1988). Inhibition of pluripotential embryonic stem cell differentiation by purified polypeptides. *Nature* 336, 688-690. 10.1038/336688a0 3143917

[DEV199477C51] Strutt, S. C., Torrez, R. M., Kaya, E., Negrete, O. A. and Doudna, J. A. (2018). RNA-dependent RNA targeting by CRISPR-Cas9. *eLife* 7, e32724. 10.7554/eLife.32724 29303478 PMC5796797

[DEV199477C52] Szczepanek, K., Chen, Q., Derecka, M., Salloum, F. N., Zhang, Q., Szelag, M., Cichy, J., Kukreja, R. C., Dulak, J., Lesnefsky, E. J. et al. (2011). Mitochondrial-targeted signal transducer and activator of transcription 3 (stat3) protects against ischemia-induced changes in the electron transport chain and the generation of reactive oxygen species. *J. Biol. Chem.* 286, 29610-29620. 10.1074/jbc.M111.226209 21715323 PMC3191002

[DEV199477C53] Taanman, J.-W. (1999). The mitochondrial genome: structure, transcription, translation and replication. *Biochim. Biophys. Acta (BBA) Bioenerg.* 1410, 103-123. 10.1016/S0005-2728(98)00161-3 10076021

[DEV199477C61] Tesoriere, A., Dinarello, A. and Argenton, A. (2021). The roles of post-translational modifications in STAT3 biological activities and functions. *Biomedicines* 9, 956. 10.3390/biomedicines9080956 34440160 PMC8393524

[DEV199477C54] Thisse, C. and Thisse, B. (2008). High-resolution in situ hybridization to whole-mount zebrafish embryos. *Nat. Protoc.* 3, 59-69. 10.1038/nprot.2007.514 18193022

[DEV199477C55] Thisse, C., Thisse, B., Schilling, T. F. and Postlethwait, J. H. (1993). Structure of the zebrafish snail1 gene and its expression in wild-type, spadetail and no tail mutant embryos. *Development* 119, 1203-1215. 10.1242/dev.119.4.1203 8306883

[DEV199477C56] Tian, Z. J. and An, W. (2004). ERK1/2 contributes negative regulation to STAT3 activity in HSS-transfected HepG2 cells. *Cell Res.* 14, 141-147. 10.1038/sj.cr.7290213 15115615

[DEV199477C57] Visavadiya, N. P., Keasey, M. P., Razskazovskiy, V., Banerjee, K., Jia, C., Lovins, C., Wright, G. L. and Hagg, T. (2016). Integrin-FAK signaling rapidly and potently promotes mitochondrial function through STAT3. *Cell Commun Signal.* 14, 32. 10.1186/s12964-016-0157-7 27978828 PMC5159999

[DEV199477C58] Wang, J., Zhou, M., Jin, X., Li, B., Wang, C., Zhang, Q., Liao, M., Hu, X. and Yang, M. (2020). Glycochenodeoxycholate induces cell survival and chemoresistance via phosphorylation of STAT3 at Ser727 site in HCC. *J. Cell Physiol.* 235, 2557-2568. 10.1002/jcp.29159 31498440

[DEV199477C59] Wegrzyn, J., Potla, R., Chwae, Y.-J., Sepuri, N. B. V., Zhang, Q., Koeck, T., Derecka, M., Szczepanek, K., Szelag, M., Gornicka, A. et al. (2009). Function of mitochondrial Stat3 in cellular respiration. *Science* 323, 793-797. 10.1126/science.1164551 19131594 PMC2758306

[DEV199477C60] Wei, W., Tweardy, D. J., Zhang, M., Zhang, X., Landua, J., Petrovic, I., Bu, W., Roarty, K., Hilsenbeck, S. G., Rosen, J. M. et al. (2014). STAT3 signaling is activated preferentially in tumor-initiating cells in claudin-low models of human breast cancer. *Stem Cells* 32, 2571-2582. 10.1002/stem.1752 24891218

