# Supplementary material for: Y705 and S727 are required for the mitochondrial import and transcriptional activities of STAT3, and for regulation of stem cell proliferation
Source: Development. 2021 Sep 6;148(17):dev199477. doi: 10.1242/dev.199477 (PMC8451946; doi:10.1242/dev.199477)
Supplement: Supplementary information [file develop-148-199477-s1.pdf]

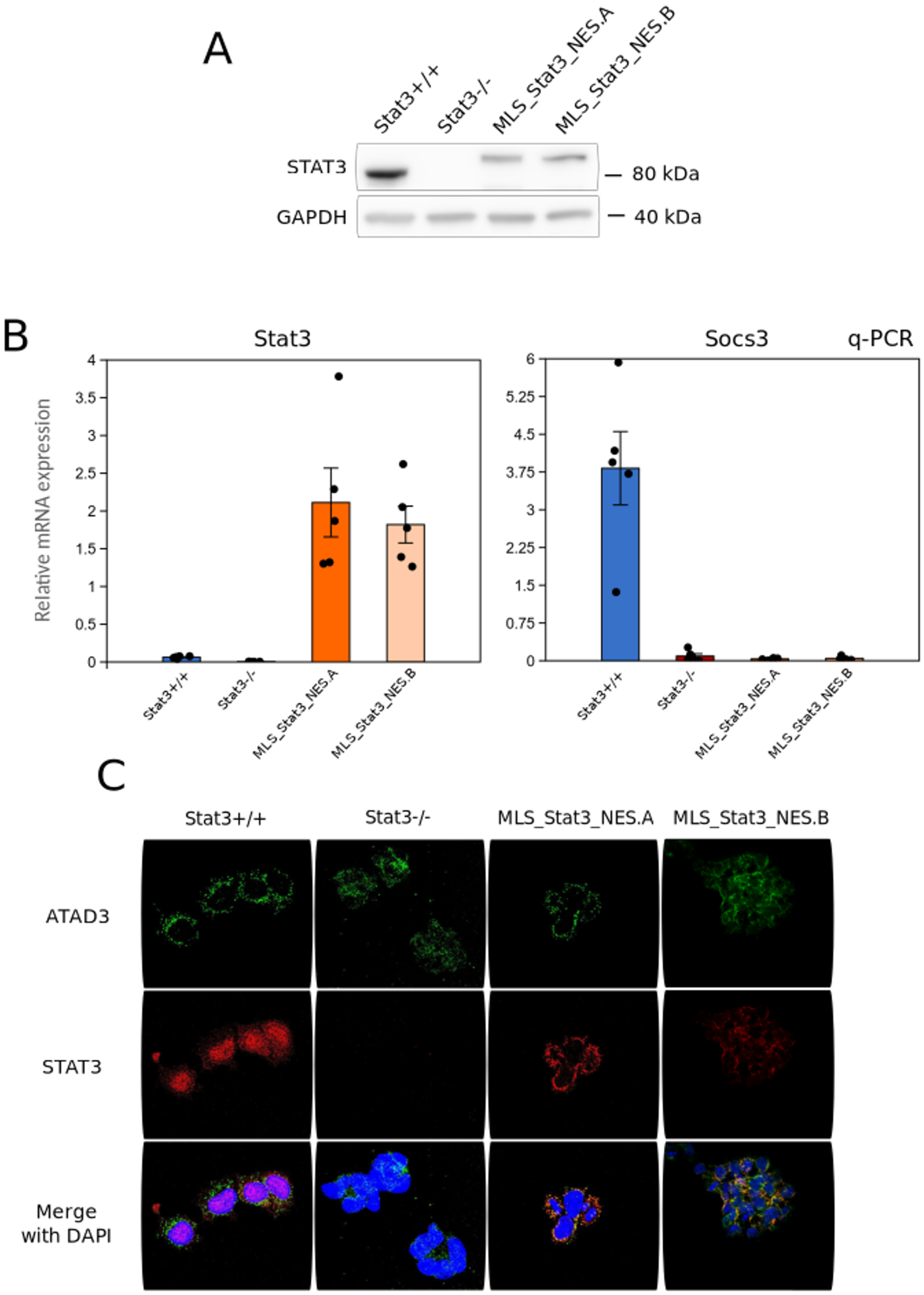

Development • Supplementary information

Fig. S1. Validation of the *MLS_Stat3_NES* construct in murine Embryonic Stem Cells. A: Western blot for total STAT3 on *Stat3*$^{+/+}$, *Stat3*$^{-/-}$ and MLS_Stat3_NES cells. Note the shift in molecular weight due to the presence of MLS and NES tags. STAT3 protein level in both MLS_Stat3_NES clones is lower than *Stat3*$^{+/+}$ cells. **B:** qPCR analysis of the Stat3 and its nuclear target gene *Socs3*. Gene expression analysis of *Stat3*$^{+/+}$ cells, *Stat3*$^{-/-}$ cells, and two MLS_Stat3_NES clones (A/B) cultured in presence of LIF. Note that both clones have the same undetectable level of *Socs3* and *Stat3*$^{-/-}$ cells. **C:** Representative confocal images of *Stat3*$^{+/+}$, *Stat3*$^{-/-}$ and MLS_Stat3_NES cells stained with anti-STAT3 and anti-ATAD3 antibodies. Merge image shows co-localization between STAT3 and the nucleoids marked by ATAD3; DAPI serves as a nuclear counterstain. Scale bar: 20 μm.

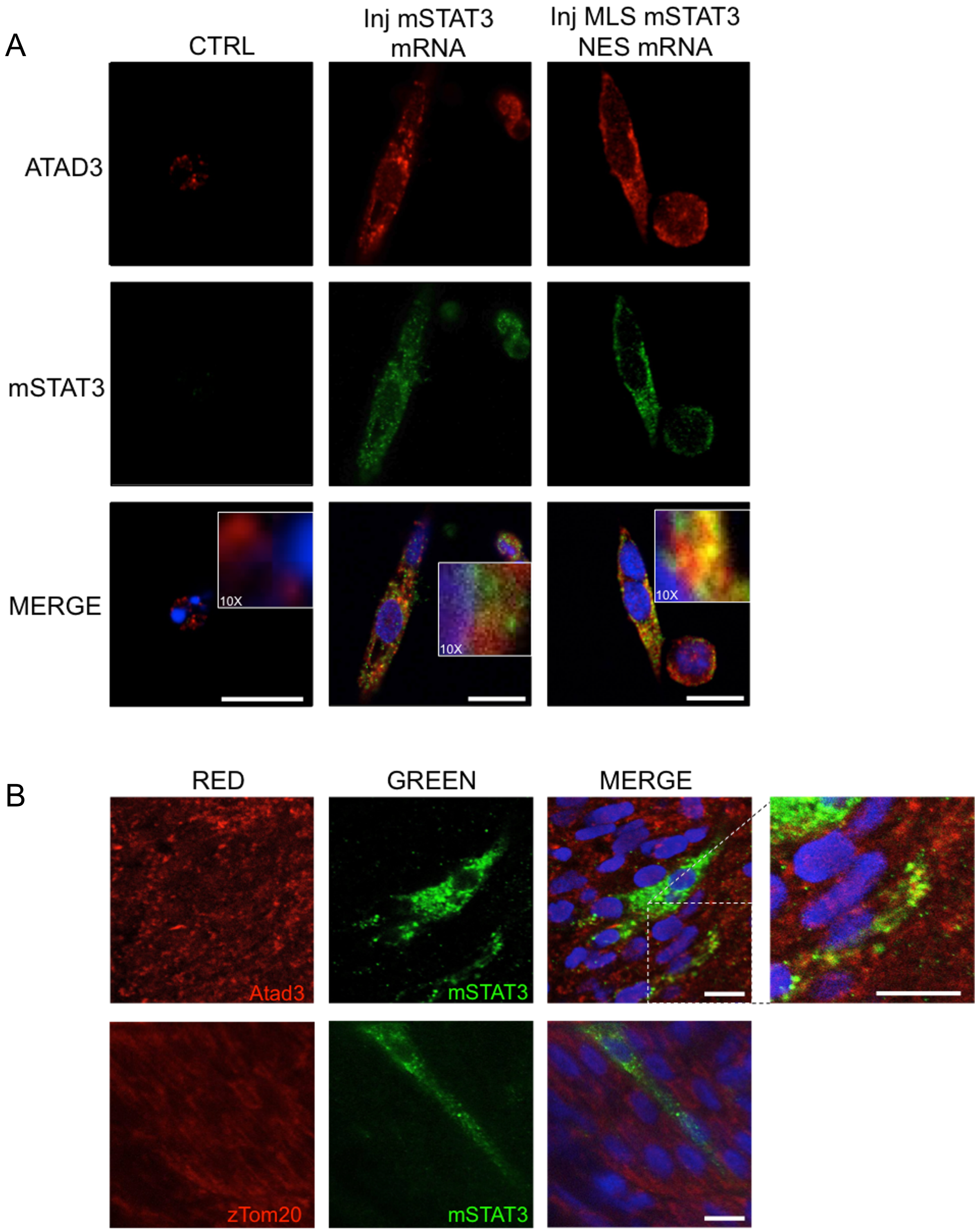
A
CTRL
Inj mSTAT3 mRNA
Inj MLS mSTAT3 NES mRNA
ATAD3
mSTAT3
MERGE
10X
10X
10X
B
RED
GREEN
MERGE
Atad3
mSTAT3
zTom20
mSTAT3

Fig. S2. Validation of the injected mRNAs on zebrafish. A: IF on zebrafish cells, dissociated and plated from 24-hpf embryos injected with *mStat3* and *MLS_mStat3* mRNA. The antibody reveals the expression of mSTAT3 (green). The mito-targeted STAT3 co-localizes with ATAD3 (red), a marker of mitochondrial nucleoids, confirming the correct subcellular localization of the proteins. Conversely the analysis of cells from embryos injected with *mStat3* mRNA results in a more diffused staining. Scale bar = 10um. **B:** Whole mount IF on 24-hpf zebrafish embryos injected with pCS2 + MLS_mSTAT3_NES plasmid. The mosaic expression is driven by a CMV promoter to verify the intracellular localization of the murine protein. mSTAT3 (green) staining confirms the expected mitochondrial localization of the protein.

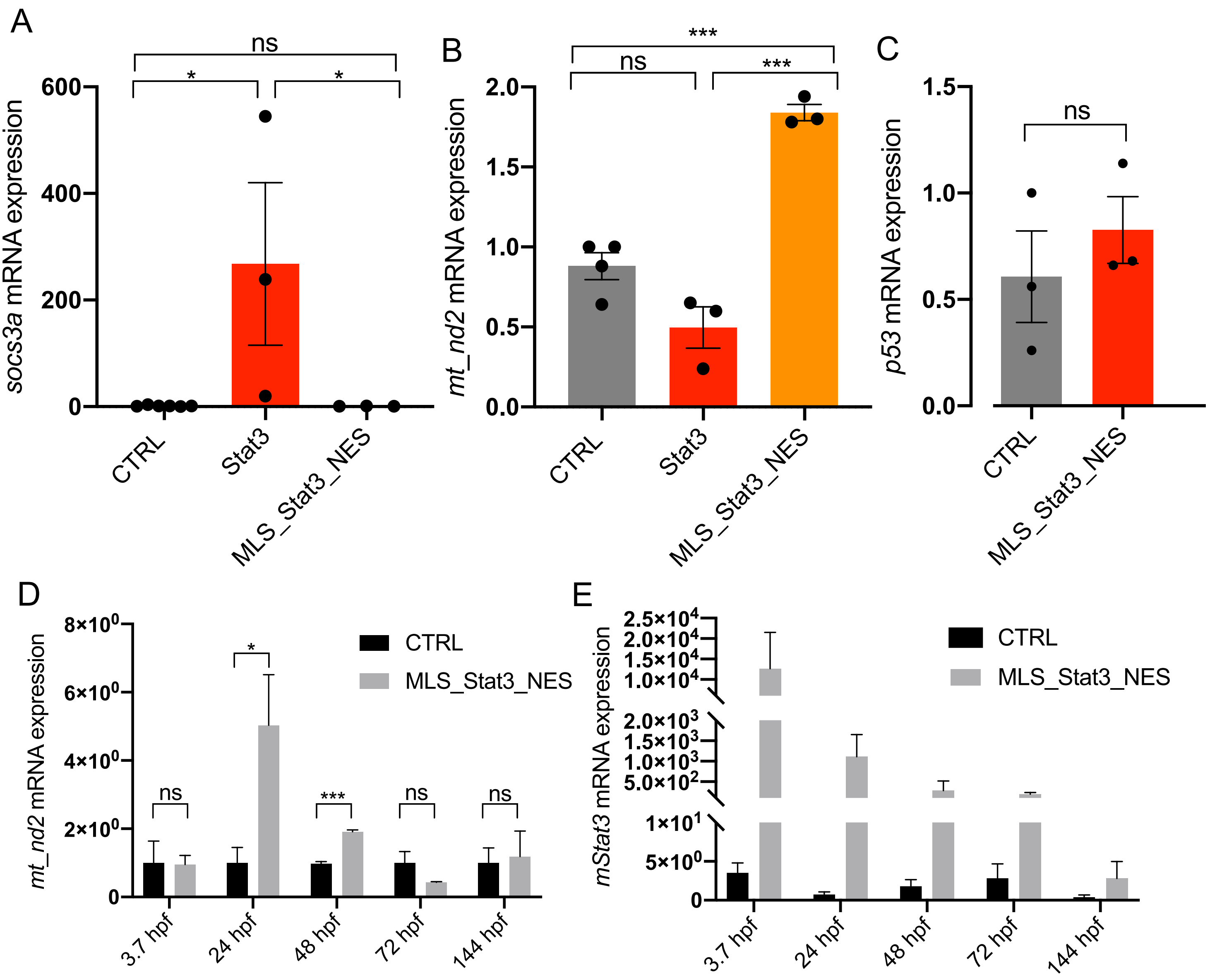

Fig. S3. Validation of effects of *mStat3* and *MLS_Stat3_NES* mRNA injected in zebrafish embryos A: qRT-PCR analysis of *socs3a* mRNA levels in 48-hpf embryos injected with *mStat3* and *MLS_mStat3_NES*. **B:** qRT-PCR analysis of *mt_nd2* mRNA levels in *mStat3* and *MLS_mStat3_NES* 48-hpf injected embryos. **C:** qRT-PCT analysis of *p53* mRNA in 48-hpf larvae injected with *MLS_mStat3_NES*. **D:** qRT-PCR analysis of *mt_nd2* levels from 3.7 hpf to 6 dpf, in larvae injected with *mStat3* mRNA. **E:** qRT-PCR analysis of *mStat3* levels from 3.7 hpf to 6 dpf, in larvae injected with *mStat3* mRNA. *bactin* was used as an internal control. Statistical analysis was performed by unpaired t-test on 3 independent biological samples (where n not specified). *p<0.05; ***p<0.001; ns=not significant; error bars=SEM.

Development • Supplementary information

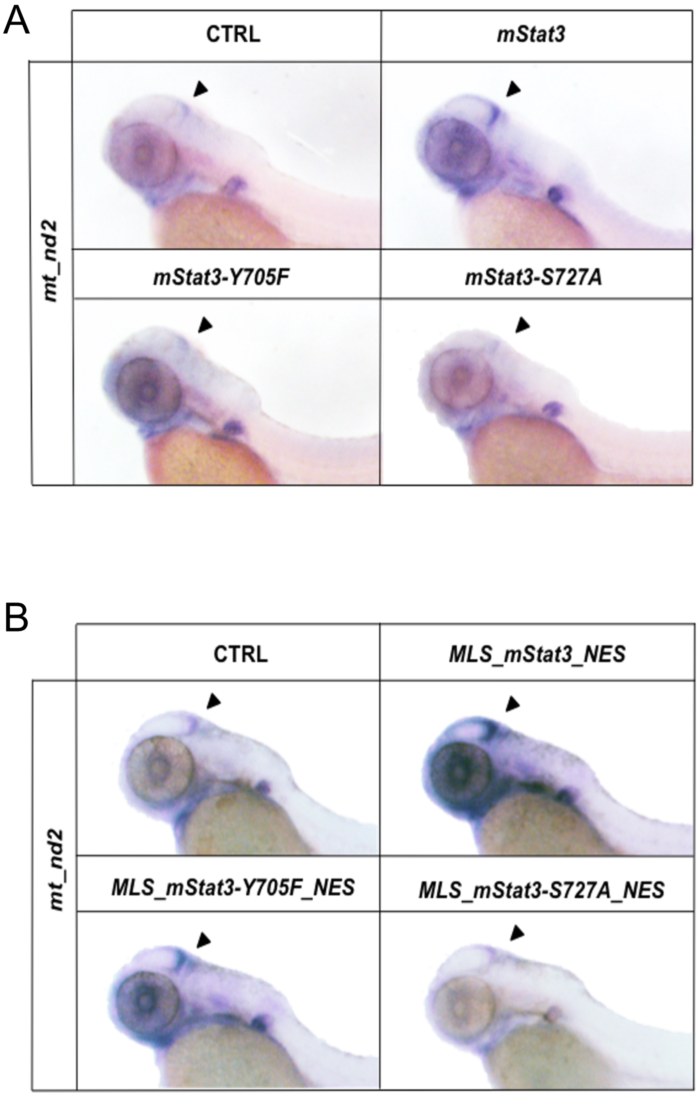

Fig. S4. STAT3-dependent mitochondrial transcription depends on Y705 and S727 phosphorylations. A: WISH with anti-*mt_nd2* mRNA probe in 48-hpf uninjected embryos (11 embryos out of 14 showed the reported signal) and embryos injected with either *mStat3* (15 embryos out of 15 showed the reported signal), *mStat3-Y705F* (17 embryos out of 18 showed the reported signal) or *mStat3-S727A* (15 embryos out of 17 showed the reported signal). **B:** WISH with anti-*mt_nd2* mRNA probe in 48-hpf uninjected embryo (24 larvae out of 24 showed the reported signal) and embryos injected with either *MLS_mStat3_NES* (21 larvae out of 31 showed the reported signal), *MLS_mStat3_NES Y705F* (19 larvae out of 20 showed the reported signal) or *MLS_mStat3_NES S727A* (17 larvae out of 21 showed the reported signal).

Development • Supplementary information

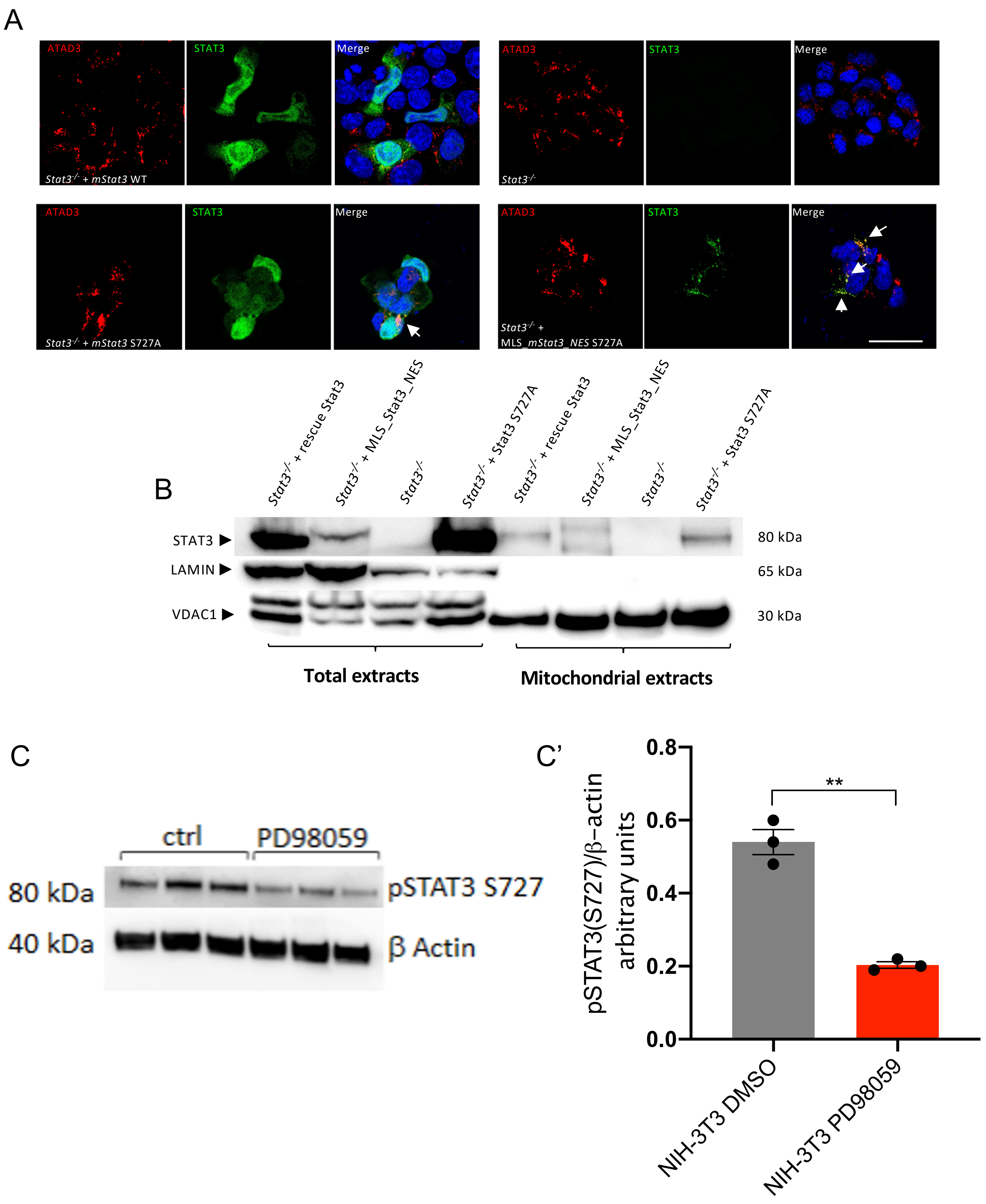
A
ATAD3
STAT3
Merge
Stat3-/- + mStat3 WT
Stat3-/-
Stat3-/- + mStat3 S727A
Stat3-/- + MLS_mStat3_NES S727A
B
Stat3-/- + rescue Stat3
Stat3-/- + MLS_Stat3_NES
Stat3-/-
Stat3-/- + Stat3 S727A
STAT3
LAMIN
VDAC1
80 kDa
65 kDa
30 kDa
Total extracts
Mitochondrial extracts
C
ctrl
PD98059
80 kDa
40 kDa
pSTAT3 S727
β Actin
C'
pSTAT3(S727)/β-actin arbitrary units
0.8
0.6
0.4
0.2
0.0
**
NIH-3T3 DMSO
NIH-3T3 PD98059

Fig. S5. Analysis of S727 modification in ESCs and in NIH-3T3 cells. A: IF with anti-STAT3 and anti-ATAD3 Ab on ESCs *Stat3*$^{-/-}$ transiently transfected with the constructs encoding: *mStat3*, *MLS_mStat3_NES, MLS_mStat3_NES S727A* or mStat3. Arrows indicate the colocalization of ATAD and STAT3. Scale bar: 200 µm. **B:** western blot of mitochondrial STAT3 from ESCs mitochondrial extracts; VDAC1 was used as a mitochondrial loading control, Lamin was used as a nuclear loading control. **C-C':** qRT-PCR analysis of *mt_nd2* and *pcna* on 48-hpf larvae treated with either PD98059 12.5 µM or DMSO. western blot analysis of pSTAT3 S727 in NIH-3T3 cells treated for 24 hours with 12.5 µM PD98059 (β-Actin was used as a loading control). Statistical analysis was performed by unpaired t-test on 3 independent biological samples. *p<0.05; error bars=SEM.

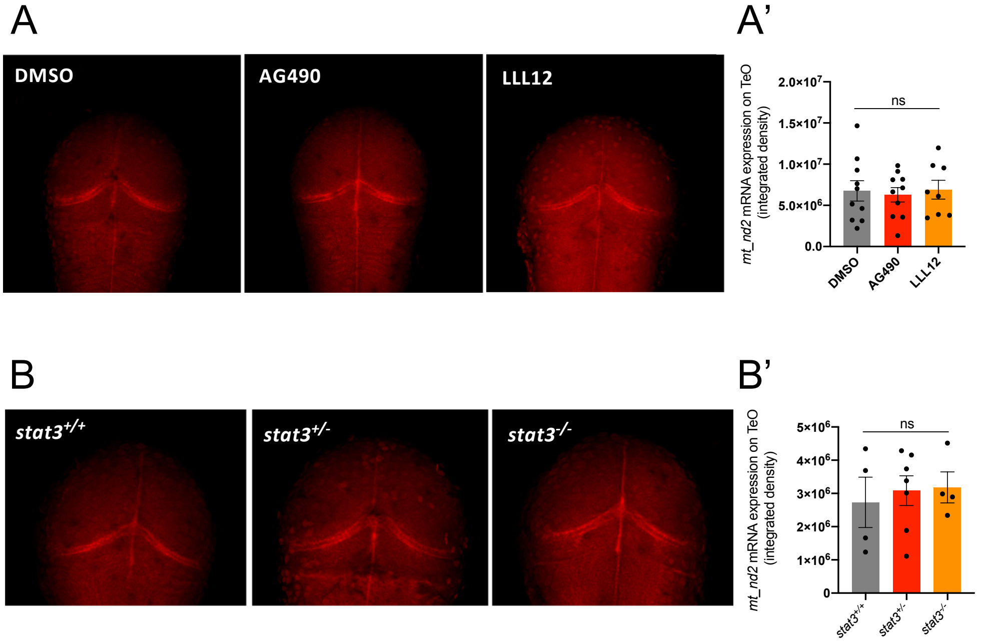

Fig. S6. *mt_nd2* mRNA expression is not affected by AG-490 nor in 48-hpf *stat3* mutant larvae. A-A': FISH with *mt_nd2* probe in the TeO of 48-hpf larvae treated for 24 hours with AG490. Fluorescence quantification of *mt_nd2* mRNA levels in the TeO (n=10). **B-B':** FISH with *mt_nd2* probe in the TeO of 48-hpf *stat3*$^{+/+}$, *stat3*$^{+/-}$, and *stat3*$^{-/-}$ larvae. fluorescence quantification of *mt_nd2* mRNA levels in the TeO. Statistical analysis was performed by unpaired t-test on 3 independent biological samples. ns = not significant; error bars=SEM.

Development • Supplementary information

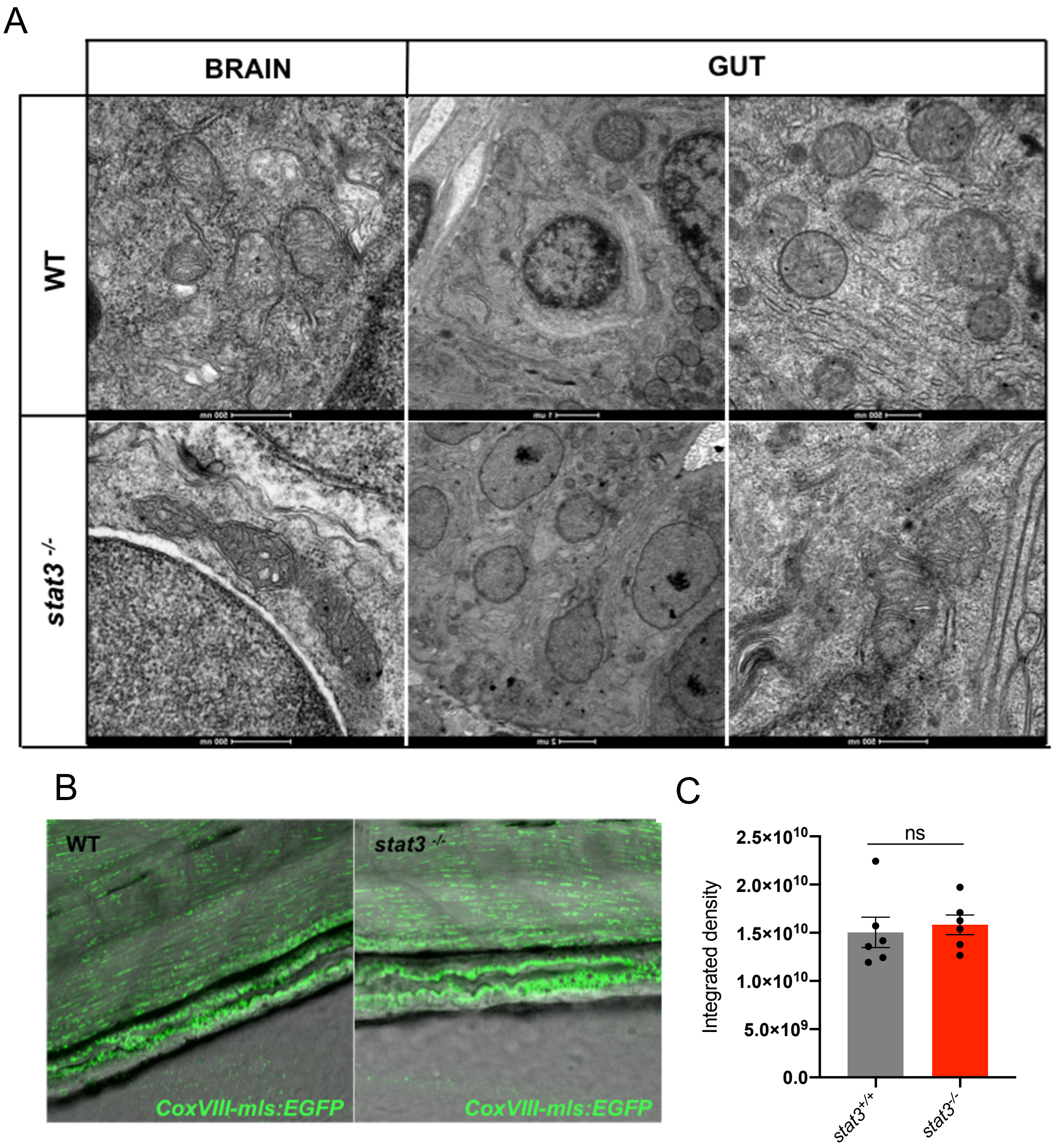

Fig. S7. Stat3 depletion does not affect mitochondria morphology and biogenesis in the brain and intestine of *stat3*$^{-/-}$ larvae. A: TEM analysis of mitochondrial morphology in intestine and brain of 6-dpf *stat3*$^{-/-}$ mutants and WT siblings. **B:** EGFP expression in the intestine of 6-dpf *stat3*$^{-/-}$/*Tg(CoxVIII-mls:EGFP)* and WT/*Tg(CoxVIII-mls:EGFP)* siblings (n=6). **C:** Fluorescence quantification of EGFP expression in the intestine of 6-dpf *stat3*$^{-/-}$/*Tg(CoxVIII-mls:EGFP)* and WT/*Tg(CoxVIII-mls:EGFP)* siblings (p-value= 0.,6878). Statistical analysis was performed by unpaired t-test on indicated number of samples*;* ns = not significant; error bars=SEM.

Development • Supplementary information

Table S1. List of sgRNAs sequences (5'-3' sequences)

Exon	Sequence	Reference
14	GGUCGAUCUUAAGUCCUUGG	Peron *et al*. (2020)
22	AGUGAGCUGCUUGGGAA	This paper
23	AUGAGAGAGUCGAGCGUGCG	This paper

Table S2. List of primer used for genotyping (5'-3' sequences)

Primer	Primer sequence	Reference
stat3 ex14 fw	GGCCTCTCTGATAGTGACCG	Peron *et al*. (2020)
stat3 ex14 rv	AGTTGTGCTTAGACGCGATC	Peron *et al*. (2020)
stat3 ex22 fw	GTGTGTGTGTTAGGCAGGCT	This paper
stat3 ex22 rv	AGCTCCCTAATGCCTACCCA	This paper
stat3 ex23 fw	TGCAGGACTAACTCTGGCAA	This paper
stat3 ex23 rv	GCTTCGTTGTGCATGAGAGA	This paper
nr3c1 fw	ACCACTTCAAGCGGACAGAG	Facchinello *et al*. (2017)
nr3c1 rv	CCGGCTTCTGATCTTTCTGC	Facchinello *et al*. (2017)

Table S3. List of cloning-related primers (5'-3' sequences)

Primer name	Primer sequence
MLS_STAT3_NES_Y705F fw	GCTGCCCCGTTCCTGAAGACC
MLS_STAT3_NES_Y705F rv	ACTACCTGGGTCGGCTTC
MLS_STAT3_NES_S727D fw	CCTGCCGATGGACCCCCGCACT
MLS_STAT3_NES_S727D rv	TCAATGGTATTGCTGCAGGTC
MLS_STAT3_NES_S727A fw	CCTGCCGATGGCCCCCCGCAC
MLS_STAT3_NES_S727A rv	TCAATGGTATTGCTGCAGGTCGTTGGTGTC
MLS_STAT3_NES_ΔDNAbd fw	GGCGATCTCCAACATCTGTCAGATGC
MLS_STAT3_NES_ΔDNAbdrv	GCGGCTGGCAAGGAGTGGGTCTC

Table S4. List of qRT-PCR and RT-PCR primers (5'-3' sequences)

Gene	Forward primer sequence	Reverse primer sequence
zmt_nd2	GCAGTAGAAGCCACCACAAA	GCTAGACCGATTTTGAGAGCC
zgapdh	GTGGAGTCTACTGGTGTCTTC	GTGCAGGAGGCATTGCTTACA
zpcna	CCTTGGCACTGGTCTTTGAA	GGCACACGAGATCATGACAG
zp53	CAACGTTGGAGCCACTTGAG	CATCTGCGGACCACTTCAG
zstat1a	GCAGCTCAAGAAACTCCTGG	AAAGGTCTCTGCAGTTGGGT
zbactin	TGGGTATGGAATCTTGCGGT	GTGGGGCAATGATCTTGATCT
zsocs3a	GGAAGACAAGAGCCGAGACT	GCGATACACACCAAACCCTG
m*Socs3*	ATTTCGCTTCGGGACTAGC	AACTTGCTGTGGGTGACCAT
m*Stat3*	TGTTGGAGCAGCATCTTCAG	GAGGTTCTCCACCACCTTCA
m*bactin*	CTAAGGCCAACCGTGAAAAG	ACCAGAGGGCATACAGGGACA

Development • Supplementary information
