# Supplementary material for: Y705 and S727 are required for the mitochondrial import and transcriptional activities of STAT3, and for regulation of stem cell proliferation
Source: Development. 2021 Sep 6;148(17):dev199477. doi: 10.1242/dev.199477 (PMC8451946; doi:10.1242/dev.199477)
Supplement: Reviewer comments [file develop-148-199477_review_history.pdf]

Y705 and S727 are required for the mitochondrial import and transcriptional activities of STAT3, and for regulation of stem cell proliferation

Margherita Peron, Alberto Dinarello, Giacomo Meneghetti, Laura Martorano, Riccardo Massimiliano Betto, Nicola Facchinello, Annachiara Tesoriere, Natascia Tiso, Graziano Martello and Francesco Argenton
DOI: 10.1242/dev.199477

Editor: Maria Elena Torres-Padilla

Review timeline

Original submission:	27 January 2021
Editorial decision:	8 March 2021
First revision received:	14 July 2021
Editorial decision:	28 July 2021
Second revision received:	30 July 2021
Accepted:	30 July 2021

Original submission

First decision letter

MS ID#: DEVELOP/2021/199477

MS TITLE: Y705 and S727 are required for mitochondrial import and transcriptional activities of STAT3 and regulate proliferation of embryonic and tissue stem cells

AUTHORS: Margherita Peron, Giacomo Meneghetti, Alberto Dinarello, Laura Martorano, Riccardo Massimiliano Betto, Nicola Facchinello, Annachiara Tesoriere, Natascia Tiso, Graziano Martello, and Francesco Argenton

Apologies for the delay in getting back to you, regarding the decision on your above manuscript, this was due to a very late report of one of our reviewers. I have now received all the referees' reports on the above manuscript, and I am pleased to report that the 3 reviewers considered your work interesting. The referees' comments are appended below, or you can access them online: please go to BenchPress and click on the 'Manuscripts with Decisions' queue in the Author Area.

As you will see, the referees express considerable interest in your work, but have some significant criticisms and recommend a substantial revision of your manuscript before we can consider publication. If you are able to revise the manuscript along the lines suggested, I will be happy to receive a revised version of the manuscript. You will see that several of the points raised are shared by the reviewers, and they suggest specific changes, amendments and experiments, which will be necessary to address in the form of a revised manuscript. In addition, reviewer 3 suggests a specific experiment to take full advantage of the in vivo potential of zebrafish, and I tend to share the reviewer's view. Also, this reviewer has concerns on the quality of the EM data provided, which are shared by Reviewer 1 upon cross-check. I thus would also strongly encourage you to improve the quality of the EM data, if you are not in the position to do so, please explain why in your response to the reviewers letter and include instead a more careful interpretation on the EM.

© 2021. Published by The Company of Biologists under the terms of the Creative Commons Attribution License (https://creativecommons.org/licenses/by/4.0/).

Your revised paper will be re-reviewed by one or more of the original referees, and acceptance of your manuscript will depend on your addressing satisfactorily the reviewers' major concerns. Please also note that Development will normally permit only one round of major revision.

We are aware that you may be experiencing disruption to the normal running of your lab that make experimental revisions challenging. If it would be helpful, we encourage you to contact us to discuss your revision in greater detail. Please send us a point-by-point response indicating where you are able to address concerns raised (either experimentally or by changes to the text) and where you will not be able to do so within the normal timeframe of a revision. We will then provide further guidance. Please also note that we are happy to extend revision timeframes as necessary.

Please attend to all of the reviewers' comments and ensure that you clearly highlight all changes made in the revised manuscript. Please avoid using 'Tracked changes' in Word files as these are lost in PDF conversion. I should be grateful if you would also provide a point-by-point response detailing how you have dealt with the points raised by the reviewers in the 'Response to Reviewers' box. If you do not agree with any of their criticisms or suggestions please explain clearly why this is so.

Reviewer 1

Advance summary and potential significance to field

This is an interesting manuscript analysing the mitochondrial function of Stat3. I think there are numerous fascinating links between mitochondria and development and stem cell function that are waiting to be explored and I really liked the in vivo analysis that was done here. Although this is an interesting manuscript I had some concerns that I think need addressing.. Unfortunately the manuscript file did not have page numbers, it hope the positions can be found from the quote or figures labels.

Comments for the author

It would be good to have a native English speaker look at the manuscript The first sentence of the intro is an overstatement….not needed

At the end of the first paragraph PCNA expression is considered fully equivalent with proliferation..I don't think this can be done without a more direct method like eg BrdU or EdU labelling.

The VVV-> AAA mutant was use to test reliance of STAT3 on DNA binding…it is a bit unfortunate that in the original report this variant was not as severly deficient in binding as an EE->AA. Why was this one not chosen?

"(Fig. 4 A,B). qRT-PCR analysis on homogenized embryos detected an increase of global mt_nd2 gene expression (Fig. 4 C,D), which .."

As it is not significant this should be rephrased to: a trend to increased expression was also noted in qRT-PCR experiments on whole embryos, but this did not reach statistical significance

In fig5 D Stat3 WT some arrows are pointing at white areas? A bit confusing, perhaps let them point at areas deemed to be positive.

…Indeed, the proliferation rate in the PML of 48-hpf embryos injected with MLS_mStat3_NES_S727A mRNA resulted significantly l..

Also

"…gene expression and cell proliferation (Fig. 6 D)."

Again in these instances, without BrdU or Edu or perhaps pH3 experiments this cannot be sure, use "expression of the proliferation marker PCNA" rather than "proliferation rate"

© 2021. Published by The Company of Biologists under the terms of the Creative Commons Attribution License (https://creativecommons.org/licenses/by/4.0/).

…mt_nd2 in stat3+/+, stat3+/-, and stat3-/- 48-hpf sibling larvae. This result is probably due to genetic compensation…

I would put a line in that stat1a has previously been seen to be upregulated in these mutants (Peron 2020)

To overcome this issue and to determine whether genetic ablation of stat3 alters mitochondrial transcription and cell proliferation in 48-hpf larvae, we decided to analyse stat3 “CRISPants" generated after injection of Cas9 protein and sgRNAs which target the antisense strand of exons 14, 22 and 23 of stat3 gene:

The current theory is that injection of these guides should also lead to compensation, thus the differential effect is a bit surprising, as they would be expected to lead nonsense mediated decay. Guides that blocks transcription of the gene would be required, can stat1a be checked in the crispants? That would help.

Figure 9a’’ could do with some clarifying labels for the lanes

Endogenous Stat3 appears to be upregulated after mStat3 injection in 9b, is this expected?

pHH3 is referred to as pH3 in another figure …make consistent It would be great if the link between abnormal mitochondrial function and proliferation could be discussed or clarified more, is there energy stress? Is AMPK activated? is P53 upregulated? It would be good to look at this, even if it is to exclude effects here.

Fig s4 some numbers are required how many were injected and looked like to picture shown eg 20/20 or 16/20…
“We provide here in vivo evidence that phosphorylation of STAT3 Y705, being required for precise mitochondrial import of STAT3, is needed for STAT3-mediated mitochondrial gene expression “ Phrasing is a bit confusing here I would phrase it as follows.

We provide here in vivo evidence that phosphorylation of STAT3 Y705, being required for precise mitochondrial import of STAT3, thus unable to induce mitochondrial gene expression. However it can function, when targeted to mitochondria using an exogenous MLS.

It would be great if a Stat3 S727 mutant could be made by gene editing…but I admit this should be a next paper.

Reviewer 2

Advance summary and potential significance to field

In this manuscript, the authors reported a novel role of mitochondrial stat3 in zebrafish development. The authors group recently reported that stat3 is required for the maintenance of intestinal stem cells (Peron et al Development, 2020). Here they assessed the function of stat3 in mitochondria using the stat3 mutant zebrafish and the mutant form of stat3 specifically localized to mitochondria. They found that the function of stat3 in mitochondria is required for intestinal cell proliferation. In addition, they revealed that the phosphorylation of Y705 and S727 are required to mediate the function in mitochondria for proper localization in mitochondria and proper transcriptional activation, respectively.

Comments for the author

The function of Stat3 in mitochondria is well analyzed in mouse embryonic stem (ES) cells (Carbognin et al EMBO J, 2016). In ES cells, it was reported that the phosphorylation of S727 is mainly mediated by FGF/MAPK (Huang et al, Stem Cells, 2014). However, there are several reports to show S727 phosphorylation by CDK8 in human CD4+ helper T cells (Martinez-Fabregas et al, Cell Rep, 2020), by mTOR in osteosarcoma cell lines (Wang et al, IUBMB Life, 2020), by TBK-1 in macrophages (Balic et al, Nat Commun, 2020), by FAK in endothelial cells (Visavadiya et al, Cell

© 2021. Published by The Company of Biologists under the terms of the Creative Commons Attribution License (https://creativecommons.org/licenses/by/4.0/).

Commun Signal, 2016) although these information were not cited in this manuscript. The authors stated that S727 in zebrafish intestine is phosphorylated by MAPK based on the result with the MEK inhibitor that interfere with the proliferation and mitochondrial transcription, but it could happen in parallel in stat3-independent manner. The role of S727 phosphorylation by TBK-1 in mouse macrophage is well documented by Balic et al (Nat Commun, 2020), in which the mouse mutant carrying S727A mutation show the defect of LPS-induced metabolic reprogramming that could be mediated by mitochondrial transcription. There are several possibilities to verify the link between MAPK and S727 such as assessing the impact of MEK inhibitor on mutant zebrafish expressing phosphomimetic mutant of S727 testing the inhibitors of CDK8, mTOR and TBK-1 to rule out their involvement, and applying other MEK inhibitor that shows higher specificity than PD98059. Direct assessment of the inhibition of S727 phosphorylation in zebrafish intestine is also required because its phosphorylation could me mediated by different pathways in cell type specific manner.

1. Page 5: the authors demonstrated that a form of mitochondrial STAT3 mutated in the DNA binding domain retains the ability to activate mitochondrial transcripts. This is interesting finding, but is there any evidence that this DNA binding domain is solely responsible to the binding to mitochondria DNA?
2. How about the function of mitochondrial STAT3 mutated in the DNA binding domain in ES cells?

3. Page 6: STAT3 Y705F mutant was first reported by Minami et al (PNAS, 1996).

4. Page 7: the proliferation rate in the PML of 48-hpf embryos injected with MLS_mStat3_NES_S727A mRNA resulted significantly lower to that of embryos injected with WT MLS_mStat3_NES mRNA---How does S727A work as a dominant negative mutant?

5. How did the authors specify JAK2 as the target of AG-490? Based on its character, JAK3 and EGFR are also possible target that mediate the biological response. How about the phenotype of Jak2, Jak3 and EGFR mutant zebrafish?

Reviewer 3

Advance summary and potential significance to field

Peron et al report the nucleus-independent role of STAT3 in regulating proliferation of progenitor cells in zebrafish. They first describe the localization of the STAT3 transcript to the area containing proliferating cells and in line with literature observe mitochondrial localization of STAT3 transcript. Using very elegant experimental tools to specifically manipulate levels of STAT3 specifically in mitochondria and not in nucleus, they could show that the proliferation of progenitors is dependent on the STAT3 level in mitochondrion. They could further identify the signalling leading to the mitochondrial localization of the STAT3. Finally, they could show that the role of STAT3 in the mitochondria is DNA-binding independent. Taken together, Peron et al do provide convincing evidences to support this rather novel mechanism of proliferation regulation by mitochondrial STAT3 and this reviewer would consider the manuscript as a strong candidate for publication in Development.

Comments for the author

Despite entire sets of experimental evidences, this reviewer would suggest to use the zebrafish in vivo model with all its advantages to address the biological meaning of this regulation. The key question that authors should try to address is what is the functional meaning of such regulation. As, it is known that STAT could be also involved in the fate commitment, it should be feasible to address if the neurogenesis in these animals is altered and the gliogenesis is prolonged. This would be essential for publishing it in Development.

In addition to such major experiment that should address the biological meaning of the regulation, several minor issues should be considered:

© 2021. Published by The Company of Biologists under the terms of the Creative Commons Attribution License (https://creativecommons.org/licenses/by/4.0/).

1. In the Fig1. authors report co-localization of the PCNA and STAT3 transcript. This should be done using two-color ISH to demonstrate expression in the same cell. Moreover, PCNA is post-transcriptionally regulated and ISH should not be used as a marker for proliferating cells. There is antibody also working in zebrafish and it should be used together with fluorescent ISH for stat3.

2. Figure 2. Data should be shown as dot-plot and not bars to allow reader to appreciate data distribution. This applies for all figures. Moreover, it is not clear what the integrated density means in panel D.

3. Figures 3, 5 and 6: Fluorescent images should be of higher resolution with orthogonal projection to demonstrate co-localization

4. Figure 5. The quality of EM images is not sufficient. It is not possible to see the localization claimed by authors.

5. Authors should consider better arrangement of panels within figures. In some Figures, plots are even overlapping.

First revision

Author response to reviewers' comments

Reviewer 1 Advance Summary and Potential Significance to Field:
This is an interesting manuscript analysing the mitochondrial function of Stat3. I think there are numerous fascinating links between mitochondria and development and stem cell function that are waiting to be explored and I really liked the in vivo analysis that was done here. Although this is an interesting manuscript I had some concerns that I think need addressing.. Unfortunately the manuscript file did not have page numbers, it hope the positions can be found from the quote or figures labels.

Reviewer 1 Comments for the Author:
It would be good to have a native English speaker look at the manuscript.
>> We revised the text and introduced page and line numbers.

The first sentence of the intro is an overstatement….not needed
>> We removed it.

At the end of the first paragraph PCNA expression is considered fully equivalent with proliferation. I don't think this can be done without a more direct method like eg BrdU or EdU labelling.
>>We test BrDU and Edu labelling, but we could not succeed in getting a clear readout of TeO. In the new Fig. 1, we performed immunofluorescence experiments against Pcna protein whose production is strictly correlated with cell proliferation. We also performed co-labelling of Pcna protein and pcna transcript and we saw a perfect overlap between these two molecules suggesting that immunofluorescence and in situ hybridization against Pcna and pcna can be both used to trace cell proliferation. Additionally, we co-stained Pcna protein with stat3 and mt_nd2 mRNAs and we saw a consistent merge between these labelling.

The VVV-> AAA mutant was use to test reliance of STAT3 on DNA binding…it is a bit unfortunate that in the original report this variant was not as severly deficient in binding as an EE->AA. Why was this one not chosen?
>> Actually, a) in the original paper (Horvath et al., 1995) where both EE->AA and VVV->AAA mutants were described, the DNA binding capacity of both mutants was equally neglectable (a faint signal in the EMSA assay);
b) the VVV->AAA mutation has been tested in other papers (Langlais et al., 2012); c) successfully introduced and validated also in STAT5 (Ilaria et al., 1999); d) in zebrafish STAT3 the VVV residues

© 2021. Published by The Company of Biologists under the terms of the Creative Commons Attribution License (https://creativecommons.org/licenses/by/4.0/).

are conserved, while the EE are not. For all these reasons we found the injection of VVV>AAA more compliant with our aims than EE>AA.

"(Fig. 4 A,B). qRT-PCR analysis on homogenized embryos detected an increase of global mt_nd2 gene expression (Fig. 4 C,D), which .." As it is not significant this should be rephrased to: a trend to increased expression was also noted in qRT-PCR experiments on whole embryos, but this did not reach statistical significance
>> We agree: we changed it according to the referee's suggestion.

In fig5 D Stat3 WT some arrows are pointing at white areas? A bit confusing, perhaps let them point at areas deemed to be positive.
>> We agree, we have ameliorated the panel, provided better pictures, inserted pointers inside each pic, and changed the description of the figure.

…Indeed, the proliferation rate in the PML of 48-hpf embryos injected with MLS_mStat3_NES_S727A mRNA resulted significantly l..
Also
"…gene expression and cell proliferation (Fig. 6 D)."
Again in these instances, without BrdU or Edu or perhaps pH3 experiments this cannot be sure, use "expression of the proliferation marker PCNA" rather than "proliferation rate"
>> We agree with the referee and we did the changes suggested.
…mt_nd2 in stat3+/+, stat3+/-, and stat3-/- 48-hpf sibling larvae. This result is probably due to genetic compensation…
I would put a line in that stat1a has previously been seen to be upregulated in these mutants (Peron 2020)
To overcome this issue and to determine whether genetic ablation of stat3 alters mitochondrial transcription and cell proliferation in 48-hpf larvae, we decided to analyse stat3 "CRISPants" generated after injection of Cas9 protein and sgRNAs which target the antisense strand of exons 14, 22 and 23 of stat3 gene:
The current theory is that injection of these guides should also lead to compensation, thus the differential effect is a bit surprising, as they would be expected to lead nonsense mediated decay. Guides that blocks transcription of the gene would be required, can stat1a be checked in the crispants? That would help.
>> We checked stat1a expression levels and, as mentioned in the text, we could not detect its upregulation in Stat3 CRISPRants (fig 9C). Hence, in this new version, we are suggesting that [these] CRISPRants do not have STAT3 genetic compensation as previously observed in stat3 knockouts (Peron et al 2020.   .
In conclusion, in our hands, CRISPRants for the stat3 gene did not show any compensation, as already observed in zebrafish also for other genes by Savage et al. (2019) and by Buglo et al. (2020). These references and observations have now been added to the manuscript.

Figure 9a'' could do with some clarifying labels for the lanes
>> We fixed the figure.

Endogenous Stat3 appears to be upregulated after mStat3 injection in 9b, is this expected?
>>The difference between CRISPRants and CRISPRants+MLS_Stat3_NES does not reach significance. These samples derive from independent pools of injected larvae. Hence, we expect some variance between samples in the expression of stat3. However, it is worth noting that socs3a, an important Stat3 target gene, is significantly downregulated both in CRISPRants and CRISPRants+MSL_Stat3_NES suggesting that in both cases Stat3 nuclear activity is severely dampened. However, in this version we rearranged the Fig. 9: A, A', A'', B, C in a more clear and straight way to represent a general characterization and validation of CRISPRant, while panels D, E, F, G show the effect upon MLS_Stat3_NES injection.

pHH3 is referred to as pH3 in another figure …make consistent
>> We did the required corrections.

It would be great if the link between abnormal mitochondrial function and proliferation could be discussed or clarified more, is there energy stress? Is AMPK activated? is P53

© 2021. Published by The Company of Biologists under the terms of the Creative Commons Attribution License (https://creativecommons.org/licenses/by/4.0/).

upregulated? It would be good to look at this ,even if it is to exclude effects here.
>>This is an interesting point that we are actively investigating in mouse ES cells. We found alterations in the levels of mitochondrial respiration and in the levels of ATP (Carbognin et al and unpublished data). We are going to investigate this point in the future. We also tested p53 expression levels in MLS_Stat3_NES larvae, but we could not see significant differences in p53 transcript level compared to uninjected larvae (Fig. S3 C).

Fig s4 some numbers are required how many were injected and looked like to picture shown eg 20/20 or 16/20…
>> We added the required information in figure legends.

"We provide here in vivo evidence that phosphorylation of STAT3 Y705, being required for precise mitochondrial import of STAT3, is needed for STAT3-mediated mitochondrial gene expression " Phrasing is a bit confusing here I would phrase it as follows.
We provide here in vivo evidence that phosphorylation of STAT3 Y705, being required for precise mitochondrial import of STAT3, thus unable to induce mitochondrial gene expression. However it can function, when targeted to mitochondria using an exogenous MLS.
>> Thanks to the referee, we have rephrased the sentence taking in great account the referee suggestion.

It would be great if a Stat3 S727 mutant could be made by gene editing…but I admit this should be a next paper.
>> We agree that generation of S727 mutants would go beyond the scope of the current study, but actually we planned to generate them to further dissect the contribution of mitochondrial Stat3.

Reviewer 2 Advance Summary and Potential Significance to Field:
In this manuscript, the authors reported a novel role of mitochondrial stat3 in zebrafish development. The authors group recently reported that stat3 is required for the maintenance of intestinal stem cells (Peron et al, Development, 2020). Here they assessed the function of stat3 in mitochondria using the stat3 mutant zebrafish and the mutant form of stat3 specifically localized to mitochondria. They found that the function of stat3 in mitochondria is required for intestinal cell proliferation. In addition, they revealed that the phosphorylation of Y705 and S727 are required to mediate the function in mitochondria for proper localization in mitochondria and proper transcriptional activation, respectively.

Reviewer 2 Comments for the Author:
The function of Stat3 in mitochondria is well analyzed in mouse embryonic stem (ES) cells (Carbognin et al, EMBO J, 2016). In ES cells, it was reported that the phosphorylation of S727 is mainly mediated by FGF/MAPK (Huang et al, Stem Cells, 2014). However, there are several reports to show S727 phosphorylation by CDK8 in human CD4+ helper T cells (Martinez-Fabregas et al, Cell Rep, 2020), by mTOR in osteosarcoma cell lines (Wang et al, IUBMB Life, 2020), by TBK-1 in macrophages (Balic et al, Nat Commun, 2020), by FAK in endothelial cells (Visavadiya et al, Cell Commun Signal, 2016) although these information were not cited in this manuscript. The authors stated that S727 in zebrafish intestine is phosphorylated by MAPK based on the result with the MEK inhibitor that interfere with the proliferation and mitochondrial transcription, but it could happen in parallel in stat3-independent manner. The role of S727 phosphorylation by TBK-1 in mouse macrophage is well documented by Balic et al (Nat Commun, 2020), in which the mouse mutant carrying S727A mutation show the defect of LPS-induced metabolic reprogramming that could be mediated by mitochondrial transcription. There are several possibilities to verify the link between MAPK and S727 such as assessing the impact of MEK inhibitor on mutant zebrafish expressing phosphomimetic mutant of S727, testing the inhibitors of CDK8, mTOR and TBK-1 to rule out their involvement, and applying other MEK inhibitor that shows higher specificity than PD98059. Direct assessment of the inhibition of S727 phosphorylation in zebrafish intestine is also required because its phosphorylation could me mediated by different pathways in cell type specific manner.
>>Thanks to the referee, we tested PD98059 in zebrafish injected with either MLS_Stat3_NES or

© 2021. Published by The Company of Biologists under the terms of the Creative Commons Attribution License (https://creativecommons.org/licenses/by/4.0/).

MLS_Stat3_NES S727D (the S727 phosphomimetic mutation): the PD98059 treatment abrogated the upregulation of mitochondrial transcription induced by MLS_Stat3_NES injection (Fig. 6B); however, PD98059 had no effects in MLS_Stat3_NES S727D injected larvae (Fig. 6D) suggesting that it is working on the MEK-ERK pathway targeting the S727. As nicely suggested by the referee, we also tested another MEK-ERK inhibitor, PD03. Unfortunately it does not have any effect on mitochondrial transcription, both in injected and uninjected larvae (panel below).

We have removed unpublished data provided for the referees in confidence.

1. Page 5: the authors demonstrated that a form of mitochondrial STAT3 mutated in the DNA binding domain retains the ability to activate mitochondrial transcripts. This is interesting finding, but is there any evidence that this DNA binding domain is solely responsible to the binding to mitochondria DNA?
2. How about the function of mitochondrial STAT3 mutated in the DNA binding domain in ES cells?
>> Thanks to the referee, this is a very interesting question, however, testing the precise specificity of the DNA binding activities of STAT3 outside of the nucleus is an entirely new program of research and it is rather orthogonal to the message of the current manuscript. In principle, we wanted to dissect, in broad terms, the involvement of the different domains of Stat3 in the induction of mitochondrial transcription. Once we discovered there is no difference in mitochondrial transcriptional activities of *MLS_Stat3_NES* and *MLS_mStat3_NES* ΔDBD, we focused on the analysis of the transactivation domain and the specific role of Y705 and S727. In this part of the paper we just wanted to show the results of our dissection: a canonical DBD does not seem to be needed for the mitochondrial activities of this protein. We are aware that these findings are opening other interesting questions, indeed we are planning to further investigate the roles of STAT3 DBD in a different project, when funded.

3. Page 6: STAT3 Y705F mutant was first reported by Minami et al (PNAS, 1996).
>>Thanks to the referee, we added Minami et al. (1996) to the bibliography.

4. Page 7: the proliferation rate in the PML of 48-hpf embryos injected with MLS_mStat3_NES_S727A mRNA resulted significantly lower to that of embryos injected with WT MLS_mStat3_NES mRNA---How does S727A work as a dominant negative mutant?
>>Actually, in the figure it is clear that S727A is not acting as a dominant active (compare with WT). If it were a DN, it would have dampened the wt signal. However, we might have missed the point raised by the refere…

5. How did the authors specify JAK2 as the target of AG-490? Based on its character, JAK3 and EGFR are also possible target that mediate the biological response. How about the phenotype of Jak2, Jak3 and EGFR mutant zebrafish?
>>The referee is right, we made a mistake as we did not really intend that JAK2 is the solely target of AG490. This is now clear in the text. AG490 is able to target all JAKs and EGFR as well, nonetheless, this does not change the interpretation of our results.

Reviewer 3 Advance Summary and Potential Significance to Field:
Peron et al report the nucleus-independent role of STAT3 in regulating proliferation of progenitor cells in zebrafish. They first describe the localization of the STAT3 transcript to the area containing proliferating cells and in line with literature observe mitochondrial localization of STAT3 transcript. Using very elegant experimental tools to specifically manipulate levels of STAT3 specifically in mitochondria and not in nucleus, they could show that the proliferation of progenitors is dependent on the STAT3 level in mitochondrion. They could further identify the signalling leading to the mitochondrial localization of the STAT3. Finally, they could show that the role of STAT3 in the mitochondria is DNA-binding independent. Taken together, Peron et al do provide convincing evidences to support this rather novel mechanism of proliferation regulation by mitochondrial STAT3 and this reviewer would consider the manuscript as a strong candidate for publication in Development.

Reviewer 3 Comments for the Author:
Despite entire sets of experimental evidences, this reviewer would suggest to use the

© 2021. Published by The Company of Biologists under the terms of the Creative Commons Attribution License (https://creativecommons.org/licenses/by/4.0/).

zebrafish in vivo model with all its advantages to address the biological meaning of this regulation. The key question that authors should try to address is what is the functional meaning of such regulation. As, it is known that STAT could be also involved in the fate commitment, it should be feasible to address if the neurogenesis in these animals is altered and the gliogenesis is prolonged. This would be essential for publishing it in Development.
>> We show here the expression analysis of sox9b, her4.3 and her5, recently demonstrated to label neuroepithelial and radial glial precursor cells in the TeO. Interestingly, no significant differences in the expression of any of the three markers was detectable in the TeO of 48hpf embryos injected with MLS_Stat3_NES with respect to uninjected controls. Moreover, we also tested whether mitoStat3 injection determines alteration of the signal in Tg(neuroD:GFP) transgenic background, but we could not observe significant alteration in fluorescent signals (not shown).The results suggest that, at least at this developmental stage, mitoStat3 is not involved in prolonging gangliogenesis or increasing the number of neural progenitors and early glial populations. Whether to include these results in the manuscript is a decision that we would leave to the referee. Our opinion is that its mention should be avoided).

We have removed unpublished data provided for the referees in confidence.

In addition to such major experiment that should address the biological meaning of the regulation, several minor issues should be considered:
1. In the Fig1. authors report co-localization of the PCNA and STAT3 transcript. This should be done using two-color ISH to demonstrate expression in the same cell. Moreover, PCNA is post- transcriptionally regulated and ISH should not be used as a marker for proliferating cells. There is antibody also working in zebrafish and it should be used together with fluorescent ISH for stat3.*>>We performed a double staining of Pcna and stat3 and we saw a partial overlapping between the two stainings (Fig. 1).*

2. Figure 2. Data should be shown as dot-plot and not bars to allow reader to appreciate data distribution. This applies for all figures. Moreover, it is not clear what the integrated density means in panel D.
>> We agree with the referee and we represent data as dot-plots.

3. Figures 3, 5 and 6: Fluorescent images should be of higher resolution with orthogonal projection to demonstrate co-localization
>> We do not think fluorescent images are informative enough at this resolution, however, they oriented our analysis inside mitochondria. These are the reasons why we decided to perform further analysis like subcellular fractionation and TEM to validate the precise localization of STAT3 mutant isoforms.

4. Figure 5. The quality of EM images is not sufficient. It is not possible to see the localization claimed by authors.
>>We fixed the panel and provided better pictures.

5. Authors should consider better arrangement of panels within figures. In some Figures, plots are even overlapping.
>> During conversion of the PDF some errors occurred. We apologise for that and we fixed the figures.

Second decision letter

MS ID#: DEVELOP/2021/199477

MS TITLE: Y705 and S727 are required for mitochondrial import and transcriptional activities of STAT3 and regulate proliferation of embryonic and tissue stem cells.

© 2021. Published by The Company of Biologists under the terms of the Creative Commons Attribution License (https://creativecommons.org/licenses/by/4.0/).

AUTHORS: Margherita Peron, Giacomo Meneghetti, Alberto Dinarello, Laura Martorano, Riccardo Massimiliano Betto, Nicola Facchinello, Annachiara Tesoriere, Natascia Tiso, Graziano Martello, and Francesco Argenton

I have now received all the referees reports on the above manuscript, and I am happy to convey that the three Reviewers found your manuscript improved. In light of their adviced, I would be happy to accept a version of your manuscript, which includes the last minor text amendments suggested by the Reviewers, including the suggestion of addinga short discussion on the findings of PD0325901, which I agree with Reviewer 2, is important for the readers who will try to reproduce the results shown in your manuscript.

The Reviewers' comments are appended below, or you can access them online: please go to BenchPress and click on the 'Manuscripts with Decisions' queue in the Author Area.

Reviewer 1

Advance summary and potential significance to field

This manuscript analyses the mitochondrial function of Stat3. There are numerous fascinating links between mitochondria and development and stem cell function that are waiting to be explored. This paper demonstrates the in vivo importance/function of two crucial amino acids of STAT3 with respect to the mitochondrial role of this gene, it convincingly shows that STAT3 regulates mitochondrial transcription.
Surprisingly STAT3 does not require its normal DNA binding domain, suggesting protein protein interactions suffice to promote trascription, indicating a new mode of action of STAT3.

Comments for the author

I am happy with the manuscript in its current form. Considering the decision on adding the extra data. This was asked for by reviewer 3, and it would be best take her/his opinion into account more than mine.
Personally, I am in favour of publishing data even if negative, but it currently feels as a story that is somewhat separate from the rest.
I had only a few tiny suggestions..in quote marks
P1 abstract: of "the" JAK/STAT3 pathway
P2 levels and on life-death cell "fate" decisions
P9 compensation, a process that "frequently" happens in zebrafish mutant
P10 I find CRISPRant unpronounceable! ….Is this official spelling? Anyway.. I prefer CRISPant but asking to change all figure labels would be excessive.

Reviewer 2

Advance summary and potential significance to field

In this revised manuscript, the authors addressed the points raised by this reviewer in proper manner. In general, the quality of the manuscript reaches to the level for publication in Development.

Comments for the author

The authors addressed the effect of PD0325901 and found that it does not have any effect on mitochondrial transcription, both in injected and uninjected larvae. This information is important for the readers who will try to reproduce the results shown in this manuscript, so it is strongly recommended to add this description in the discussion part with explanation of the possible reason of this unexpected result.

Reviewer 3

© 2021. Published by The Company of Biologists under the terms of the Creative Commons Attribution License (https://creativecommons.org/licenses/by/4.0/).

Advance summary and potential significance to field

Authors properly addressed all my concerns.

Comments for the author

Authors properly addressed all my concerns.

Second revision

Author response to reviewers' comments

The suggestion of including negative results into discussion (and introduce the relative references) has been taken into account.

Third decision letter

MS ID#: DEVELOP/2021/199477

MS TITLE: Y705 and S727 are required for mitochondrial import and transcriptional activities of STAT3 and regulate proliferation of embryonic and tissue stem cells.

AUTHORS: Margherita Peron, Alberto Dinarello, Giacomo Meneghetti, Laura Martorano, Riccardo Massimiliano Betto, Nicola Facchinello, Annachiara Tesoriere, Natascia Tiso, Graziano Martello, and Francesco Argenton

Thank you for revising your manuscript to incorporate the last minor suggestions from our Reviewers. I am happy to tell you that your manuscript has been accepted for publication in Development, pending our standard ethics checks.

© 2021. Published by The Company of Biologists under the terms of the Creative Commons Attribution License (https://creativecommons.org/licenses/by/4.0/).
